# Abundantly expressed class of noncoding RNAs conserved through the multicellular evolution of dictyostelid social amoebas

**DOI:** 10.1101/gr.272856.120

**Published:** 2021-03

**Authors:** Jonas Kjellin, Lotta Avesson, Johan Reimegård, Zhen Liao, Ludwig Eichinger, Angelika Noegel, Gernot Glöckner, Pauline Schaap, Fredrik Söderbom

**Affiliations:** 1 Department of Cell and Molecular Biology, Uppsala University, Uppsala S-75124, Sweden;; 2 Department of Molecular Biology, Biomedical Center, Swedish University of Agricultural Sciences, Uppsala S-75124, Sweden;; 3 Department of Cell and Molecular Biology, National Bioinformatics Infrastructure Sweden, Science for Life Laboratory, Uppsala University, Uppsala S-75124, Sweden;; 4 Centre for Biochemistry, Institute of Biochemistry I, Medical Faculty, University of Cologne, 50931 Cologne, Germany;; 5 College of Life Sciences, University of Dundee, Dundee DD1 5EH, United Kingdom

## Abstract

Aggregative multicellularity has evolved multiple times in diverse groups of eukaryotes, exemplified by the well-studied development of dictyostelid social amoebas, for example, *Dictyostelium discoideum*. However, it is still poorly understood why multicellularity emerged in these amoebas while the majority of other members of Amoebozoa are unicellular. Previously, a novel type of noncoding RNA, Class I RNAs, was identified in *D. discoideum* and shown to be important for normal multicellular development. Here, we investigated Class I RNA evolution and its connection to multicellular development. We identified a large number of new Class I RNA genes by constructing a covariance model combined with a scoring system based on conserved upstream sequences. Multiple genes were predicted in representatives of each major group of Dictyostelia and expression analysis confirmed that our search approach identifies expressed Class I RNA genes with high accuracy and sensitivity and that the RNAs are developmentally regulated. Further studies showed that Class I RNAs are ubiquitous in Dictyostelia and share highly conserved structure and sequence motifs. In addition, Class I RNA genes appear to be unique to dictyostelid social amoebas because they could not be identified in outgroup genomes, including their closest known relatives. Our results show that Class I RNA is an ancient class of ncRNAs, likely to have been present in the last common ancestor of Dictyostelia dating back at least 600 million years. Based on previous functional analyses and the presented evolutionary investigation, we hypothesize that Class I RNAs were involved in evolution of multicellularity in Dictyostelia.

The role of RNA goes far beyond being an intermediate transmitter of information between DNA and protein in its role as messenger (m)RNA. This has been appreciated for a long time for some noncoding RNAs (ncRNAs), such as transfer (t)RNAs, ribosomal (r)RNAs, small nuclear (sn)RNAs, and small nucleolar (sno)RNAs. Today, we know that ncRNAs are involved in regulating most cellular processes, and the advent of high-throughput sequencing technologies have facilitated the identification of numerous different classes of ncRNAs (Cech and Steitz 2014). These regulatory RNAs vary greatly in size from 21–24 nt, for example, micro (mi)RNAs and small interfering (si)RNAs, to several thousands of nucleotides, such as long noncoding (lnc)RNAs. Several classes of ncRNAs are ubiquitously present in all domains of life, but others are specific to certain evolutionary linages, contributing to their specific characteristics. This can be exemplified by Metazoa, in which an increase in the number of ncRNAs, for example, miRNAs, is associated with increased organismal complexity and is believed to have been essential for the evolution of metazoan multicellularity (Gaiti et al. 2017).

Multicellularity in plants and animals is achieved by clonal division and development originating from a single cell. This is in contrast to aggregative multicellularity, were cells stream together to form multicellular structures upon specific environmental changes. Aggregative multicellularity has evolved independently multiple times and is found both among eukaryotes and prokaryotes (Lasek-Nesselquist and Katz 2001; Brown et al. 2009, 2011, 2012a,b; He et al. 2014; Tice et al. 2016; Kawabe et al. 2019). The complexity of the aggregative multicellular life stages varies for different organisms, but they all share the transition from unicellularity to coordinated development upon environmental stress, such as starvation, which eventually leads to formation of fruiting bodies containing cysts or spores (Kawabe et al. 2019). Probably the most well-studied aggregative multicellularity is the development of the social amoeba *Dictyostelium discoideum* belonging to the group Dictyostelia within the supergroup Amoebozoa. Dictyostelia is a monophyletic group estimated to date back at least 600 million years (Heidel et al. 2011), which is similar to the age of Metazoa (dos Reis et al. 2015). Dictyostelia is currently divided into four major groups (Group 1–4) in which all members share the ability to transition from uni- to multicellularity upon starvation (Schilde et al. 2019). However, the complexity of the development and the morphology of the fruiting bodies varies between different dictyostelids, in which the highest level of multicellular complexity is found among Group 4 species, which includes *D. discoideum* (Romeralo et al. 2013; Schilde et al. 2014). Recently a new taxonomy was proposed for many dictyostelids (Sheikh et al. 2018). As this new taxonomy has not yet been fully adopted by the research community, we choose to use the previous designations throughout this study (old and new names, including NCBI accession numbers, are summarized in Supplemental Table S1).

Well-annotated genome sequences are available for representative species of all four major groups of Dictyostelia (Eichinger et al. 2005; Heidel et al. 2011; Sucgang et al. 2011; Urushihara et al. 2015; Glöckner et al. 2016), and multiple draft genome sequences are available for additional dictyostelids. This has allowed for comparative genomics, which has provided information about protein-coding genes that are important for the diversification of Dictyostelia from other amoebozoans. Comparison between genomes has also given insight into the genes required for the evolution of the distinct morphological characteristics, which define each group (Eichinger et al. 2005; Heidel et al. 2011; Sucgang et al. 2011; Glöckner et al. 2016; Hillmann et al. 2018). However, evolution of complex traits such as multicellularity in Dictyostelia as well as other eukaryotic groups, cannot solely be explained by the appearance of novel genes but also relies on an increased ability to regulate preexisting genes and their products so that they can function in novel genetic networks (Glöckner et al. 2016; Deline et al. 2018). This is also supported by the major transcriptional reprogramming during multicellular development in *D. discoideum* (Rosengarten et al. 2015).

*D. discoideum* harbors several classes of developmentally regulated ncRNAs with regulatory potential, for example, microRNAs (Hinas et al. 2007; Avesson et al. 2012; Meier et al. 2016; Liao et al. 2018), long noncoding RNAs (Rosengarten et al. 2017), and long antisense RNAs (Hildebrandt and Nellen 1992; Rosengarten et al. 2017). In addition, a large part of the ncRNA repertoire of *D. discoideum* is constituted by Class I RNAs, originally identified in full-length cDNA libraries (Aspegren et al. 2004). So far, Class I RNAs have only been experimentally validated in *D. discoideum* (Aspegren et al. 2004; Avesson et al. 2011), but they have also been computationally predicted in *Dictyostelium purpureum* (Sucgang et al. 2011). Both species belong to the same evolutionary group of Dictyostelia, that is, Group 4 (Sheikh et al. 2018; Schilde et al. 2019). In *D. discoideum,* Class I RNAs are 42–65 nt long and are expressed at high levels from a large number of genes. Members of Class I RNAs are characterized by a short stem structure, connecting the 5′ and 3′ ends, and a conserved 11-nt sequence motif adjacent to the 5′ part of the stem. The remainder of the RNA is variable both in sequence and structure (Fig. 1A). Class I RNAs mainly localize to the cytoplasm (Aspegren et al. 2004), where one of the RNAs has been shown to associate with four different proteins of which at least one, the RNA recognition motif (RRM) containing protein Rnp1A (also known as Cibp), directly binds to the Class I RNA (Avesson et al. 2011). Furthermore, the Class I RNAs appear to be involved in regulating multicellular development. This is based on the observations that Class I RNAs are developmentally regulated and that cells in which a single Class I RNA gene has been knocked out show aberrant early development (Aspegren et al. 2004; Avesson et al. 2011).

**Figure 1.**
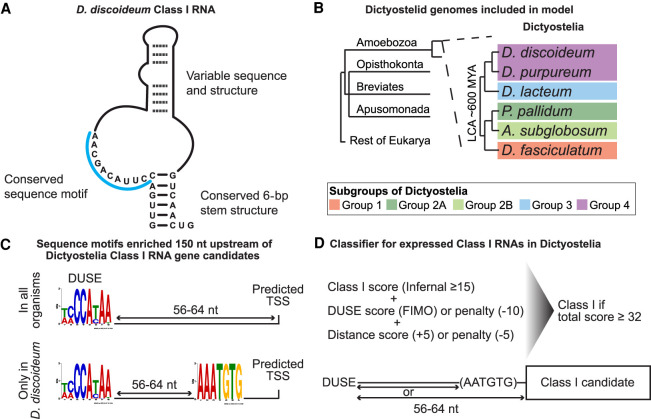
Search strategy and classification of Class I RNAs. (*A*) Schematic representation of previously described *D. discoideum* Class I RNAs (Avesson et al. 2011). (*B*) Schematic phylogeny showing the location of Amoebozoa, a sister group to Obazoa (Opisthokonta, Breviates, and Apusomonada) in the eukaryotic tree of life based on Burki et al. (2020). Dictyostelia is represented by species belonging to each major group (Schilde et al. 2019). The genomes of these dictyostelids were searched for Class I RNA genes, and newly identified genes were used to refine the covariance model. (*C*) Enriched sequence motifs identified upstream of Class I RNA gene candidates in the different dictyostelids represented in *B* (Infernal score ≥ 25, *n* = 126). The putative promoter motif (DUSE) is found ∼60 nt from the predicted start of transcription (TSS) in all organisms (*upper*). DUSE in combination with TGTG box, only identified in *D. discoideum* (*lower*). (*D*) Summary of scoring system used for the classifier of Dictyostelia Class I RNA based on Infernal score ≥ 15, presence of DUSE, and distance between DUSE and predicted TSS or TGTG box.

In this study we used computational approaches combined with experimental verification to investigate the prevalence of Class I RNAs within Dictyostelia as well as in other organisms with the overall aim to understand if Class I RNAs are restricted to dictyostelids and perhaps associated with aggregative multicellularity.

## Results

### Covariance model identifies Class I RNA genes in evolutionarily distinct groups of Dictyostelia social amoebas

The presence of Class I RNA genes in two different dictyostelids and the fact that at least one Class I RNA member is involved in controlling early multicellular development (Aspegren et al. 2004; Avesson et al. 2011; Sucgang et al. 2011), led us to hypothesize that this class of ncRNA may be a general effector for early development in all members of dictyostelid social amoebas. To investigate this, we used the complete and well-annotated genome sequences for representatives of each major group of Dictyostelia, that is, *D. discoideum* (Eichinger et al. 2005), *D. purpureum* (Sucgang et al. 2011), *Dictyostelium lacteum* (Glöckner et al. 2016)*, Polysphondylium pallidum* (Heidel et al. 2011)*, Acytostelium subglobosum* (Urushihara et al. 2015), and *Dictyostelium fasciculatum* (Fig. 1B; Heidel et al. 2011). Class I RNAs cannot be reliably detected by sequence searches alone owing to the high sequence variability. Therefore, we constructed a covariance model (CM) with Infernal (Nawrocki and Eddy 2013) in which both sequence and secondary structure information of 34 *D. discoideum* Class I RNAs were taken into account (for details, see Methods). The initial CM search of the six Dictyostelia genomes, followed by manual inspection of the results, indicated the presence of Class I RNAs in all major groups of Dictyostelia (Supplemental Fig. S1). These candidates scored ≥25 in the CM search and had the potential to form a short stem similar to the *D. discoideum* Class I RNAs. To improve the CM, these candidates (CM score ≥ 25 and potential to form stem) were added to the CM followed by new genome searches. This process was repeated until no new candidates fulfilling the criteria were identified, after which a final search with increased sensitivity was performed (Methods). In total, 126 loci distributed over all major groups of Dictyostelia were identified (Supplemental Fig. S1), including 36 of the 40 published *D. discoideum* Class I RNAs (Aspegren et al. 2004; Avesson et al. 2011) and all the 26 previously predicted *D. purpureum* Class I loci (Sucgang et al. 2011).

### Refining the search for Class I RNA genes using conserved promoter elements

Many *D. discoideum* ncRNA genes have an upstream putative promoter element, DUSE (*Dictyostelium* upstream sequence element), which in most cases is situated ∼60 nt from the transcriptional start site (TSS) (Hinas and Söderbom 2007). However, for *D. discoideum* Class I RNAs, DUSE is often found further upstream. In these cases, a TGTG box (AAATGTG) is located ∼60 nt downstream from DUSE, and the distance from the start of the mature RNA varies. Whether the TGTG box is an additional promotor element or the TSS of a precursor transcript is currently not known. DUSE appears to be conserved within Group 4 of Dictyostelia, because it was also identified ∼60 nt upstream of the predicted *D. purpureum* Class I RNAs (Sucgang et al. 2011). To investigate the presence of conserved upstream motifs in the rest of Dictyostelia, we searched for motifs within 150 nt upstream of all the 126 Class I RNA gene candidates identified in the CM search. By this approach, DUSE-like motifs could be identified ∼60 nt upstream of the predicted start for the majority of the Class I RNA gene candidates (73 of 126) in all organisms. In contrast, the TGTG box was only found in a subset (21 of 126) of the upstream sequences, all of which belonged to *D. discoideum* Class I RNA genes (Fig. 1C). Because both sequence and distance of DUSE appeared to be conserved in all major groups of Dictyostelia, we used this information to create a scoring system, called Class I classifier, anticipating accurate prediction of expressed Class I RNA genes (Fig. 1D). First the score produced by the CM search (Infernal) was used, and all candidates with a score ≥ 15 were included to capture more divergent Class I RNA genes. Next, we scored the presence and location of DUSE and TGTG box within 150 nt upstream of the candidates identified in the CM search based on the motif identification program FIMO (Grant et al. 2011). Lack of DUSE and/or noncanonical distance from predicted TSS or TGTG box was penalized with negative scores (Methods). Taken together, a total score of 32 could be achieved if a high-scoring DUSE was identified at the predicted distance upstream of a candidate Class I RNA gene with the lowest allowed Infernal score (≥15). Based on this, all candidates scoring 32 or higher were classified as Class I RNA loci. Using this approach, we predicted 18–39 Class I RNAs (146 in total) for each of the six dictyostelids investigated.

### Class I RNAs of predicted sizes are expressed at high levels in all four groups of Dictyostelia

**Figure 2.**
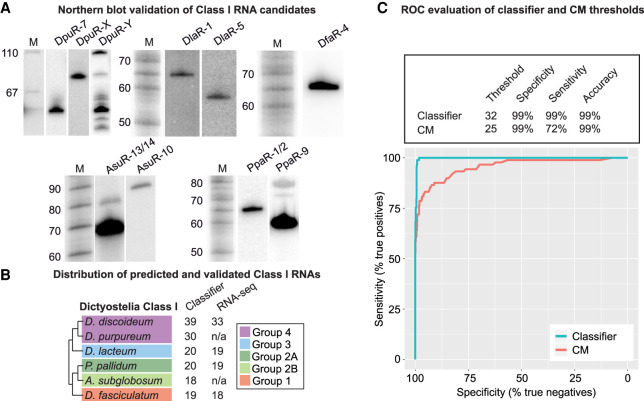
Expression of predicted Class I RNA genes. (*A*) Northern blot validation of different Class I RNAs from *D. purpureum* (DpuR), *D. lacteum* (DlaR), *D. fasciculatum* (DfaR), *A. subglobosum* (AsuR), and *P. pallidum* (DpaR). The number after the species-specific designations indicates which Class I RNA the probe recognizes. When two numbers are given, the probe recognized two different Class I RNAs. DpuR-X indicates that the probe is expected to hybridize to six different Class I RNAs predicted to be 85 nt long. DpuR-Y indicates that the probe is expected to hybridize to 24 Class I RNAs. For each organism except for *D. fasciculatum* (DfaR), the same membrane was probed, stripped, and reprobed for the different Class I RNAs. Radioactively labeled size marker is indicated by M, and numbers to the *left* indicate sizes in nucleotides. (*B*) Number of Class I RNA genes in each species according to the classifier. RNA-seq designate the number of expressed Class I RNA genes verified by RNA-seq. (*C*) ROC curves based on the RNA-seq validation and either classifier score or CM score for all Class I candidates in *D. discoideum*, *D. lacteum*, *P. pallidum*, and *D. fasciculatum* identified in the CM search. Input data are available in Supplemental Table S2. Evaluation of the classifier and CM thresholds used throughout the study are shown *above* the plot. Individual ROC curves for each organism are found in Supplemental Figure S3.

Based on the Class I classifier, we predicted Class I RNA genes in all dictyostelids included in the CM build. But are all these genes really expressed and how accurate are the size predictions? From our previous studies, we know that Class I RNAs in *D. discoideum* are expressed at high levels at vegetative growth and are readily detected by northern blot (Aspegren et al. 2004; Avesson et al. 2011). Hence, we used the same approach to validate a subset of randomly chosen candidates with a classifier score ≥ 32 in *D. purpureum* (Group 4), *D. lacteum* (Group 3), *P. pallidum* (Group 2A), *A. subglobosum* (Group 2B), and *D. fasciculatum* (Group 1). RNA was prepared from vegetative growing amoebas, and specific Class I RNAs were analyzed by northern blot (Fig. 2A). For *D. purpureum*, we probed for DpuR-7, predicted to be 54 nt long, resulting in a strong signal. In addition, we designed two probes predicted to recognize six different 85-nt-long RNAs (DpuR-X) and the majority (24/30) of Class I RNAs (DpuR-Y), respectively. As expected, probing for DpuR-X resulted in one band; for DpuR-Y, we could detect several distinct bands within the expected size range. In *D. fasciculatum*, we probed for one Class I RNA predicted to be 62 nt long (DfaR-4) while at least two candidates were probed for in the other organisms, that is, *D. lacteum*: DlaR-1 (61 nt) and DlaR-5 (54 nt); *P. pallidum*: PpaR-1/2 (59/62 nt) and PpaR-9 (58 nt); and *A. subglobosum*: AsuR-13/14 (61 nt) and AsuR-10 (82 nt). Distinct bands were detected for each Class I RNA candidate and the sizes matched the predictions well although the northern results often indicated that the RNAs were a few nucleotides longer than predicted (Fig. 2A; and see below). The larger (but much weaker) bands observed for AsuR-13/14 and PpaR-9 are likely cross hybridizations to longer Class I RNAs. Taken together, the results confirm that Class I RNAs are conserved and expressed in all four groups of Dictyostelia social amoebas.

### Classifier accurately predicts expressed Class I RNAs in all major groups of Dictyostelia

Next, we performed RNA-seq on *D. discoideum*, *D. lacteum*, *P. pallidum,* and *D. fasciculatum* representing each major group of Dictyostelia. RNA was prepared from growing cells as well as two multicellular life stages, that is, mound and slug/finger stages, to increase our chances to also detect Class I RNAs that are only expressed at specific life stages. Expression was evaluated based on the read count and coverage over all loci identified in the CM search (Infernal score ≥ 15) as exemplified in Supplemental Figure S2. RNA-seq confirmed expression for almost all Class I RNA candidates identified by the classifier (classifier score ≥ 32) both during vegetative growth and development (Fig. 2B; Supplemental Table S2). The expression data also allowed us to investigate sensitivity and specificity of our computational search approaches. For this, we calculated receiver operating characteristic (ROC) curves to evaluate the classifier performance and investigate if it improves Class I RNA identification compared to CM search alone. ROC curves were generated for *D. discoideum*, *D. lacteum*, *P. pallidum,* and *D. fasciculatum* individually (Supplemental Fig. S3) as well as for the pooled data (Fig. 2C) based on the RNA-seq validation and either CM search score (≥25) or classifier score (≥32). Evaluation of the two search approaches show an increase in both sensitivity and accuracy of prediction for the classifier, that is, when the promoter (DUSE) presence and distance were included in the classification of Class I RNA gene candidates. To summarize, the classifier reliably detects expressed Class I RNAs in all the tested dictyostelids with almost no false positives.

### Conserved features of Dictyostelia Class I RNA

The wealth of newly identified Class I RNA genes in six evolutionarily separated dictyostelids allowed us to construct a general/unifying picture of Class I RNAs. This will also be of importance when searching for Class I RNA genes in other species to track down the birth of this class of ncRNAs in evolution (see below).

#### Class I transcription is dependent on DUSE

**Figure 3.**
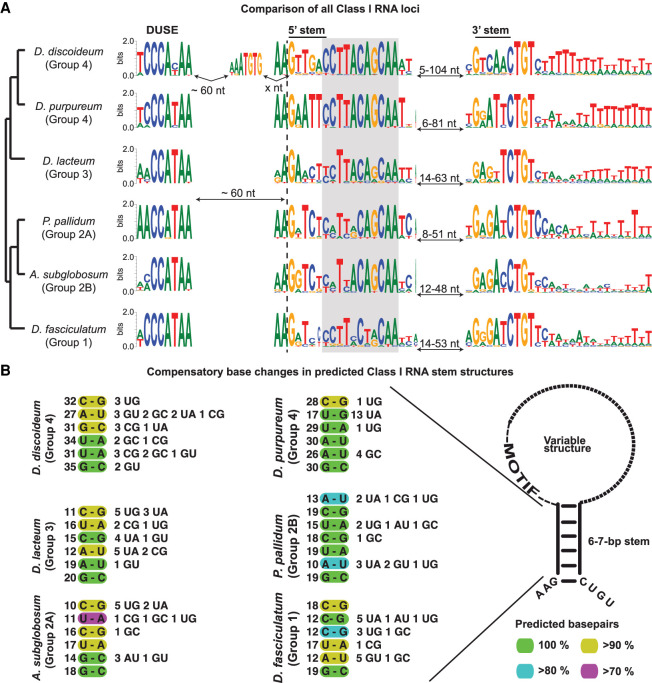
Conserved characteristics of Class I RNAs. (*A*) Sequence logo representing species-specific alignments of conserved features of Class I RNA loci. DUSE indicates the putative promoter element, and the 5′ and 3′ stem sequences of the conserved stem structure are indicated. The conserved 11-nt sequence motif adjacent to the 5′ part of the stem is boxed in gray. The dashed vertical line denotes the predicted start of the Class I RNAs based on the CM search. The sequence logo between DUSE and the 5′ stem motif for *D. discoideum* represent the TGTG box. Numbers of nucleotides correspond to the distances between indicated motifs. (*B*) Displayed are nucleotides representing the most common base pair for each position (numbers to the *left*) of the conserved stem structure for each organism (color key down *right*). Numbers to the *right* represent less common nucleotide combinations predicted to base pair. The few combinations of nucleotides not predicted to base pair are not shown. Schematic structure of Class I RNA is presented to the *right*.

Both the sequence of DUSE and its upstream location is highly conserved in all of the analyzed amoebas (Fig. 3A). In Groups 1 and 4 dictyostelids, DUSE contains three consecutive C residues, whereas two consecutive Cs are found in the majority of the DUSE of *A. subglobosum* (Group 2B) and *D. lacteum* (Group 3) and in all *P. pallidum* DUSE (Group 2A). The RNA-seq data strongly indicate that this putative promoter element is essential for transcription because it is found in front of all expressed Class I RNA genes. Further strengthening its importance is the observation that high-scoring Class I RNAs (both considering score produced by classifier or CM alone) lacking DUSE at the correct upstream location are not expressed (Supplemental Table S2). The TGTG box, situated 60 nt downstream from the DUSE and upstream of the predicted TSS, is only found in *D. discoideum,* suggesting that this is a rather late addition in the evolution of Class I RNA genes.

#### 5′ and 3′ ends of Class I RNAs are conserved

The RNA-seq analyses showed that the *D. discoideum* Class I RNAs started at the predicted (and previously defined) (Aspegren et al. 2004) G residue, but the majority of Groups 1–3 Class I RNAs started 1–2 nt upstream of the conserved G (Supplemental Fig. S2). When we compared all loci, we noticed that the 2 nt preceding the completely conserved G residue, are highly conserved A-residues in all six species investigated. The difference in 5′ ends of the mature Class I RNAs indicates that either transcription initiation or 5′ processing of Class I RNAs differ between Group 4 species and species belonging to the other groups of Dictyostelia. Also, the very 3′ end of Class I RNAs is highly similar with an almost perfectly conserved CTGT sequence in the genomic loci. The coverage from the RNA-seq data indicates that these 4 nt are transcribed so that the CUGU sequence is included in the mature Class I RNA (Supplemental Fig. S2), where the C residue always has base-pairing potential with the conserved 5′ G residue (Fig. 3A,B). The RNA-seq coverage agrees with the slightly longer than predicted lengths of Class I RNAs observed by northern blot (see above).

#### Class I RNA GC content, size, sequence motif, and stem structure are conserved throughout Dictyostelia

Comparison of all identified Class I RNAs showed that both Class I RNA length (median of ∼60 nt) and GC content (32%–41%) are highly conserved (Supplemental Fig. S4A,B). The stable GC content of Class I RNAs is remarkable considering the variation in overall genome GC content for these six dictyostelids (Supplemental Fig. S4B). In spite of these conserved features and specific sequence motifs discussed above and later, the overall sequence variability of Class I RNAs is extensive both within and between species. Only a few examples of loci with identical sequences are found within *D. discoideum*, *D. purpureum,* and *P. pallidum*, respectively (Supplemental Table S2). No Class I genes with identical sequences were found between these six analyzed species.

In contrast to the overall variable sequences, the 11 nt sequence motif identified among the *D. discoideum* Class I RNAs is highly conserved both within and between all six dictyostelids (marked in gray in Fig. 3A). The motif is nearly perfectly conserved within Group 4, whereas some positions of the motif are variable in Groups 1–3. However, T, C, C, A, and A at position 3, 6, 9, 10, and 11 (counting from the 5′-most nucleotides of the motif) are almost identical between all the Class I RNA genes regardless of species. Other nucleotides are well conserved in most of the evolutionary groups but not all. The sequence motif does not seem to extensively engage in base-pairing because computational prediction indicates that the conserved motif is less structured compared to the full-length RNA in most of the organisms (Supplemental Fig. S4C,D). However, the first 5′ nucleotide of the motif is often part of the stem structure (see below), but the base-pairing potential for the remainder of the sequence drops in a pattern similar for all six dictyostelids (Supplemental Fig. S4E).

Another distinct feature common to all *D. discoideum* Class I RNAs is the short (6 bp) stem structure, connecting the 5′ and 3′ ends of the RNA (Fig. 3A). This stem is predicted to be present in all Class I RNAs in all six species. However, in contrast to the conserved sequence motif, the nucleotide sequence of the stem structure has changed substantially during Dictyostelia evolution. Nevertheless, the base-pairing potential is retained, indicating that it is the structure rather than sequence that is crucial for function (Fig. 3B). This is further supported by the high number of compensatory mutations found in the predicted stem of Class I RNAs within each species (Fig. 3B). In spite of the sequence variation in the stems, the 5′-most G is completely conserved within all Class I RNAs from all six dictyostelids representing each evolutionary group of Dictyostelia. The predicted base-paired structure of the stem and the unstructured feature of the conserved sequence motif correspond well with previous in vitro probing results of one Class I RNA, DdR-21, from *D. discoideum* (Avesson et al. 2011). Taken together, Class I GC content, length, stem structure, and 11-nt motif are highly conserved in all evolutionary groups of Dictyostelia, indicating that these parts are essential for Class I RNA function.

### Class I RNAs are developmentally regulated and highly conserved throughout Dictyostelia

We knew from our previous work that *D. discoideum* Class I RNAs are developmentally regulated and that cells in which the gene for the Class I RNA DdR-21 has been disrupted are disturbed in early development, leading to more and smaller fruiting bodies compared to wild-type cells (Avesson et al. 2011). To investigate if the developmental regulation is conserved also in other dictyostelids, we performed principal component analysis (PCA) of Class I expression in *D. discoideum*, *P. pallidum,* and *D. fasciculatum* based on the RNA-seq data. *D. lacteum* was not included in this analysis because only one replicate per time point was available. The PCA plots show developmental regulation of Class I RNAs in all three amoebas as the different life stages are clearly separated (Supplemental Fig. S5). Taken together, this suggests a role for Class I RNAs in regulating multicellular development in Dictyostelia.

**Figure 4.**
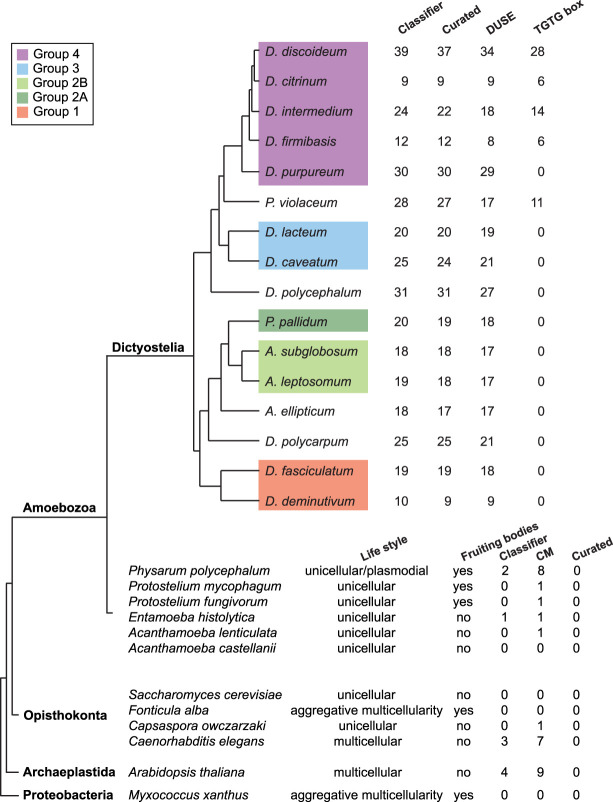
Class I RNAs are ubiquitous in and restricted to dictyostelid social amoebas. (*Upper*) Class I RNA loci were searched for in the genomes of 16 different Dictyostelia (Supplemental Table S1). The number of hits identified by the classifier is indicated as well as the number of these loci that passed manual curation. The number of curated loci with DUSE and the TGTG box at the correct distance are shown. (*Bottom*) Result from searches for Class I RNA loci outside Dictyostelia. Lifestyle indicates unicellular or multicellular organisms. Fruiting bodies denotes if the organism lifestyle involves formation of fruiting bodies. CM indicates number of candidates identified with a CM score ≥ 25. Headings Classifier and Curated as described above. Further information about outgroup Class I RNA candidates is available in Supplemental Table S4.

If the prediction that Class I RNAs are involved in and important for multicellular development holds true, these ncRNAs should be present in all dictyostelids. To analyze this, we used the Class I classifier to investigate the presence of Class I RNA genes in 10 additional social amoebas genome sequences. Class I RNA genes were detected in all species, 9–31 genes in each genome, of which the great majority passed manual curation based on the ability to form a short stem connecting the 5′ and 3′ end (Fig. 4). It should be noted that these are draft genome sequences of varying degree of completeness (Supplemental Table S1). Comparison of all curated loci reinforced the previously identified conserved Class I features, that is, the high sequence conservation of the terminal residues and the 11-nt motif as well as the short stem where structure but not sequence is preserved. In addition, presence of DUSE ∼60 nt upstream of the majority of the identified Class I RNA genes strongly suggests that expressed Class I RNAs exist in all members of Dictyostelia. The TGTG box, previously only found upstream of *D. discoideum* Class I RNA loci, was identified in four additional genomes all belonging to Group 4 or the *P. violaceum* complex, strengthening the hypothesis that this motif emerged rather late in Class I evolution. In addition, both Class I RNA lengths and GC content are conserved also when considering all species (Supplemental Fig. S6). Taken together, this proves the existence and emphasizes the importance of Class I RNA genes throughout the evolution of Dictyostelia social amoebas. We have named all curated Class I RNA genes according to the naming convention previously defined for *D. discoideum* Class I RNA genes (Supplemental Table S3; Aspegren et al. 2004).

### Genomic distribution of Class I RNA genes

In *D. discoideum*, all Class I RNA genes are located in intergenic regions and frequently found in clusters of two or more genes (Hinas and Söderbom 2007). The intergenic location also applies to the other dictyostelid species and clusters of at least two (different) Class I RNA genes are present in all analyzed Dictyostelia genomes, except for *D. citrinum* (Supplemental Fig. S7). The absence of Class I RNA gene clusters in *D. citrinum* is likely a consequence of the quality of the genome assembly (Supplemental Table S1), which is also reflected in the low number of identified Class I RNA genes. Although many Class I RNA genes cluster together, they rarely have identical sequences. Only a few species-specific identical Class I RNAs were found in *D. polycephalum*, *P. violaceum*, *D. purpureum*, *P. pallidum,* and *D. discoideum*, where in the latter two the identical genes are located in clusters (Supplemental Fig. S7). Hence, copies of identical Class I RNA genes are present within different dictyostelid, but are there also Class I RNAs that are identical between two different species? The only examples found were two Class I RNA loci shared between the Group 4 species *D. discoideum* and *D. firmibasis* (Supplemental Table S3).

To explore the origin of Class I RNAs further, we used the most well-annotated genomes to search for shared synteny by identifying orthologous genes in the 10-kb region flanking each Class I RNA locus (Methods). Using this approach, we did not identify any strong evidence for shared synteny for Class I RNA genes between the different groups of Dictyostelia. Next, we investigated if shared synteny could be detected within Group 4 only by performing the same search using the genomes of *D. discoideum*, *D. purpureum,* and *D. firmibasis*. Almost half of the Class I RNAs in *D. firmibasis* appear to share synteny with *D. discoideum* Class I RNAs, supported by several protein gene orthologs (Supplemental Fig. S8), but no well-supported examples were found for *D. purpureum*. For the identical Class I RNA genes in *D. discoideum* and *D. firmibasis*, shared synteny was detected for DfiR-4 and DdR-47 (Supplemental Fig. S8). Shared synteny between the other two identical Class I RNAs, DfiR-12 and DdR-50, could not be properly assessed because of the lack of available genome sequence surrounding DfiR-12.

### Class I RNAs are unique to dictyostelid social amoebas

The omnipresence of Class I RNAs within Dictyostelia, their developmental regulation, as well as the aberrant development of *D. discoideum* cells lacking DdR-21 (Avesson et al. 2011) led us to hypothesize that this class of ncRNAs might be involved in the evolution of Dictyostelia aggregative multicellularity. To investigate this further, we searched for Class I RNA genes in genomes of unicellular amoebas and amoebas able to form unicellular fruiting bodies. Furthermore, we explored representative genomes of other major eukaryotic groups, that is, Archaeplastida and Opisthokonta. We also chose to include the proteobacteria *Myxococcus xanthus* because these bacteria show aggregative multicellularity (Muñoz-Dorado et al. 2016), which in many aspects are analogous to Dictyostelia multicellularity (Fig. 4). We searched these genomes using the same successful approaches as for Dictyostelia, that is, using the Class I classifier, based on promoter characteristics combined with RNA structure and sequence, as well as CM search alone. Only a few candidates were identified with the Class I classifier. This was anticipated because we did not expect the DUSE sequence or its distance to the TSS to be conserved outside Dictyostelia. The Infernal search resulted in a slightly higher number of Class I RNA gene candidates. However, manual inspection revealed that the candidates are unlikely to represent true Class I RNA genes because they were few in numbers and did not share characteristics, such as conserved 5′ and 3′ ends and presence of conserved sequence motif (Fig. 4; Supplemental Table S4). Taken together, no Class I RNA genes were identified outside Dictyostelia, suggesting that this class of ncRNAs is unique to dictyostelid social amoebas and important for their aggregative multicellularity.

### Conserved Class I RNA interacting protein

Class I RNAs are conserved throughout the evolution of Dictyostelia but does this also apply to proteins associated with this class of ncRNA? We previously identified four Class I RNA interacting proteins in *D. discoideum*. One of these, Rnp1A (Ngo et al. 2016), harbors two RNA recognition motifs (RRMs) and binds directly to the Class I RNA DdR-21 (Avesson et al. 2011). Orthologs for *rnp1A* were found in all Dictyostelia genomes investigated except for *D. citrinum*, likely owing to the quality of the genome assembly (Supplemental Table S1). Furthermore, mRNA-seq data from *D. discoideum* (Group 4) (Parikh et al. 2010) as well as *D. lacteum* (Group 3), *P. pallidum* (Group 2), and *D. fasciculatum* (Group 1) (Glöckner et al. 2016) showed that *rnp1A* gene expression during early development is regulated in a way similar to that of Class I RNAs in *D. discoideum* (Supplemental Fig. S9A; Avesson et al. 2011). The majority of the orthologs are predicted to encode an approximately 300 amino acids (aa) long protein in which the N- and C-terminal sequences contain RRMs, but the central part of the protein is less conserved (Supplemental Fig. S9B). We do not know how Class I RNA and Rnp1A functionally interact; despite several efforts by us and others, all attempts to generate a *rnp1A* knockout strain in *D. discoideum* have been unsuccessful, indicating that Rnp1A is essential (www.remi-seq.org; Avesson et al. 2011; Ngo et al. 2016).

## Discussion

The development of high-throughput sequencing techniques has led to the discovery of numerous ncRNAs. In particular, it has facilitated the identification of small and long ncRNAs. However, “midsized” ncRNA have largely been overlooked partly because of the size selection commonly carried out before sequencing to enrich for small RNAs and to avoid abundant RNAs such as rRNAs and tRNAs or fragments thereof. Here, we used genome analyses in combination with expression validation to prove the existence of a class of midsized ncRNAs, Class I RNAs, in a large number of dictyostelid social amoeba. Our analysis indicates that Class I RNAs were present in the last common ancestor of Dictyostelia, dating back at least 600 million years, and we hypothesize that they were involved in the transition from unicellular to multicellular life.

Class I RNAs play an important role in *D. discoideum*, as suggested by, for example, the large number of highly expressed genes and the requirement for the Class I RNA DdR-21 for normal multicellular development (Aspegren et al. 2004; Avesson et al. 2011). This class of ncRNAs was initially discovered by sequencing cDNA libraries of full-length RNA sized 50–150 nt (Aspegren et al. 2004). Later, different bioinformatic approaches were used to predict Class I RNA genes. Fragrep, a tool that predicts ncRNAs based on sequence motifs separated by a variable region, identified 45 Class I RNA candidate genes in *D. discoideum,* of which 34 had been previously experimentally validated (Aspegren et al. 2004; Mosig et al. 2006). Another method, which searched for enriched 8-mers in the genome sequence downstream from the putative promoter element DUSE, predicted 26 Class I RNA genes in another Group 4 dictyostelid, *D. purpureum* (Sucgang et al. 2011). To investigate if Class I RNAs are present in all dictyostelids as well as in other organisms outside Dictyostelia, we first constructed a Class I RNA covariance model, which uses conserved sequence and structure motifs to predict Class I RNA genes. To increase the specificity and sensitivity, we created a classifier that evaluates the candidates identified with the covariance model based on the presence of DUSE at the correct distance from TSS or from the TGTG box (found upstream of many *D. discoideum* Class I RNA genes). With this approach, we predicted a large number of Class I RNA genes in all available genomes of Dictyostelia. Northern blot and RNAs-seq analyses of selected dictyostelids confirmed expression of the great majority of the predicted Class I RNA genes.

**Figure 5.**
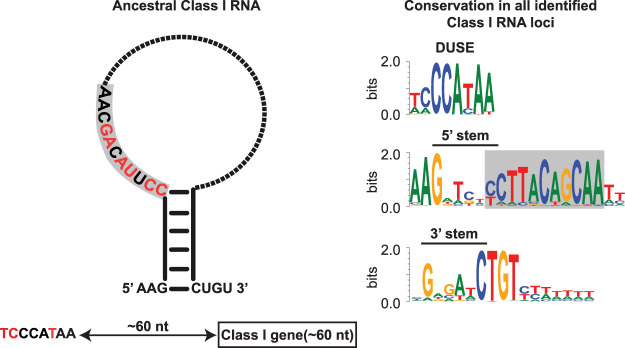
Ancestral Class I RNA and conserved key features. (*Left*) Schematic representation of the ancestral Class I RNA transcript. The putative promoter element DUSE and its distance to the Class I RNA gene is indicated *below* the Class I RNA structure. Strongly conserved features, nucleotides and base-paired stem, are colored black, whereas red denotes more variable positions (based on data presented in Fig. 3). The dotted part of the loop indicates highly variable sequence. (*Right*) Sequence logos of alignments of conserved sequence motifs from all identified and curated Class I RNAs. Only DUSE identified at the correct distance were included in the alignment. The 11-nt motif is boxed in gray in both the ancestral Class I RNA (*left*) and sequence logo (*right*).

The high number of novel Class I RNA loci identified in Dictyostelia enabled comparative studies, which provides information on their key features. The short stem connecting the 5′ and 3′ ends of mature Class I RNA is conserved. Furthermore, the sequence variability of the stem between organisms but also the high number of compensatory mutations within each species strongly suggest that it is the structure rather than sequence that is important for function. Flanking the stem structure are highly conserved nucleotides constituting the start, AAG, and end, CUGU, of the Class I RNAs. In contrast to other dictyostelids, Class I RNAs in *D. discoideum* almost always start with the G residue. Another conserved feature is the ∼11-nt motif present adjacent to the 5′ part of the stem structure. Although some of the nucleotides in this motif vary within and in between organisms, several residues are nearly perfectly conserved. The putative promoter motif, DUSE, situated ∼60 nt upstream of the start of transcription (or from the TGTG box) is highly conserved. Based on studies of spliceosomal RNAs in *D. discoideum*, we previously showed that DUSE is associated with genes transcribed by both RNA polymerase II (Pol II) and III (Pol III) (Hinas et al. 2006). However, the Class I RNA genes may be transcribed by Pol III because the canonical Pol III termination signal (a run of consecutive Ts) (Richard and Manley 2009) is present downstream from many Class I RNAs. Taken together, we conclude that Class I RNAs were present in the last common ancestor of Dictyostelia. Our data also strongly suggest that the putative promoter element DUSE was present 60 nt upstream of the ancestral Class I RNA gene and that the element was required for expression of the gene. We also conclude that the ancient Class I RNA was characterized by a short stem structure and an 11-nt sequence motif, where at least five of the positions were identical to the corresponding nucleotides in extant Class I RNAs. Owing to the high sequence and structure variability of the region between the 11-nt motif and the start of the 3′ stem in identified Class I RNAs, we cannot resolve this part of the ancestral sequence. However, the total length of the mature RNA was probably ∼60 nt long (Fig. 5).

Next, we asked if Class I RNAs are specific to Dictyostelia or if these ncRNAs are present also in other organisms. We applied our search approaches on different genomes from a battery of evolutionary diverse organisms representing species with strict unicellular lifestyles as well as those that go through different kinds of multicellular development. We did not find any Class I RNA genes in these organisms, not even in the closest unicellular relatives *Physarum polycephalum*, *Protostelium mycophagum*, or *Protostelium fungivorum* (Fig. 4). This suggests that these ncRNAs are restricted to dictyostelid social amoebas, were present in the last common ancestor of Dictyostelia, and may have played a role in the transition from unicellular life to aggregative multicellularity. This is further supported by the developmental regulation of Class I RNAs, and that at least one member, DdR-21, is required for normal development (Aspegren et al. 2004; Avesson et al. 2011). In *D. discoideum*, transition from unicellular growth to multicellular development is associated with large transcriptional reprogramming of protein-coding genes (Rosengarten et al. 2015), and different cell types (i.e., prespore and prestalk cells) can be separated based on the transcriptional signatures of individual cells (Antolović et al. 2019). Furthermore, the majority of the protein-coding genes that are essential for multicellular development in *D. discoideum* are also present in strictly unicellular amoebas (Glöckner et al. 2016). Hence, temporal and spatial regulation of the expression of these genes has played a major role in the evolution of multicellular development. Maybe Class I RNAs can rewire gene expression of genes present in unicellular organisms to create new networks adapted for development. This could be orchestrated by Class I RNA binding directly to target mRNAs to regulate gene expression. Another possibility would be that Class I RNAs regulate development by binding to proteins that directly or indirectly control development, maybe by acting as a molecular sponge, where specific proteins are sequestered by Class I RNAs. This could buffer the action of these proteins. Perhaps the observed down-regulation of Class I RNAs during development lead to an increase in free active proteins important for multicellular development. *Rnp1A* would be a candidate for this kind of regulation because Rnp1A directly interacts with Class I RNAs, at least with the tested DdR-21, in *D. discoideum* (Avesson et al. 2011). As expected for a protein important for multicellular development of social amoebas, orthologs of the *rnp1A* gene could be identified in all analyzed dictyostelids with genome assemblies of good quality. Putative *rnp1A* orthologs were identified when genomes of organisms outside Dictyostelia were included in the search (Supplemental Table S5). The tendency to differentially regulate genes to create new functions is seen also in metazoan evolution, where an increase in regulatory miRNAs is correlated with increased organismal complexity (Gaiti et al. 2017). *D. discoideum* is one of the few organisms outside animals and land plants where miRNAs have been identified (Hinas et al. 2007; Avesson et al. 2012; Meier et al. 2016; Liao et al. 2018). Whether miRNAs, similar to Class I RNAs, are present in other dictyostelids is currently being investigated.

In Dictyostelia, the most complex multicellularity is found within Group 4, which is exemplified by regulated proportions of specialized cell types and a migrating slug stage (Kawabe et al. 2019). In analogy to miRNA expansion in complex animals, the number of expressed Class I RNA genes is correlated with Dictyostelia complexity, in which Group 4 has the largest number of Class I RNA genes (Fig. 2B). In addition, RNA-seq data indicate that Class I RNAs are expressed at higher levels in Group 4 (*D. discoideum*), further strengthening that Class I RNAs are involved in increased organismal complexity. This enhanced expression appears to be connected to the TGTG box between DUSE and the TSS. So far, the TGTG box has only been identified in Class I RNA loci in species belonging to Group 4 (except *D. purpureum*) and *P. violaceum* complex (Figs. 3A, 4; Supplemental Table S3). Thus, this motif is likely a rather late addition in the evolution of Class I RNAs. It is currently not known if the second motif is actually a promoter element or the TSS of a longer precursor that is processed to the mature RNA. In either case, the TGTG box is associated with Class I RNA genes in social amoebas with higher levels of complexity as compared to other dictyostelids and appears to add another layer of regulation to Class I RNA expression. The emergence of the TGTG box somewhere after the split of Groups 3 and 4 and its connection to increased phenotypic complexity seen in Group 4 dictyostelids is somewhat analogous to changes in *cis*-regulatory elements, such as enhancers, and morphological evolution in animals (Gaunt and Paul 2012).

Although some motifs are highly conserved within all Class I RNAs, conservation of complete Class I RNA genes are rare. Only a few examples of identical loci within the same genome are found in a handful of dictyostelids and only two identical Class I RNAs shared by two different species were identified, that is, the Group 4 dictyostelids *D. discoideum* and *D. firmibasis* (Supplemental Table S3). In *D. discoideum* and *P. pallidum,* many Class I RNA genes are situated in larger clusters, and it is within these clusters where the species-specific identical Class I RNA genes are found, perhaps indicating expansion of Class I RNA genes by duplication. The snRNA genes in *D. discoideum* are organized in a similar way in that closely related genes often are found in pairs situated very close together (Hinas and Söderbom 2007). However, in general the Class I RNA genes are spread out in the genomes of the different dictyostelids. Hence, the low occurrence of shared synteny, low overall sequence conservation, and different number of loci in different organisms suggest that the expansion of Class I RNAs mainly occurred after speciation.

In conclusion, we have identified Class I RNAs in 16 different dictyostelids and validated their expression in representatives of each major group of Dictyostelia dating back approximately 600 million years. Despite the large evolutionary distances, Class I RNA genes share upstream motifs (putative promoters), and the mature RNAs have several characteristics in common, that is, short stem, conserved sequence motif, and highly conserved 5′ and 3′ ends. In addition, the *D. discoideum* Class I RNA interacting protein Rnp1A is conserved throughout Dictyostelia. Although Class I RNAs are present in all dictyostelids investigated, no evidence was found for this class of RNAs in any other organism, including the closest sequenced true unicellular relatives *P. polycephalum*, *P. mycophagum,* and *P. fungivorum.* Taken together, experimental data show that Class I RNAs are developmentally regulated, and we previously showed that depletion of Class I RNA DdR-21 leads to aberrant early multicellular development. These observations, together with the finding that Class I RNA genes are present in all dictyostelids investigated, possibly implies that this class of RNA was involved in the evolution of multicellularity in Dictyostelia.

## Methods

### RNA isolation and northern blot

The following strains were used for northern blot validation of Class I expression: *D. discoideum* AX2 (DBS0235521, www.dictybase.org), *D. purpureum* WS321, *P. pallidum* PN500, *D. lacteum* Konijn, *D. fasciculatum* SH3, *A. subglobosum* LB1. All strains, except *D. discoideum*, were kindly provided by Dr. Maria Romeralo and Professor Sandra Baldauf. Total RNA was extracted with TRIzol (Thermo Fisher Scientific) from cells grown in association with *Klebsiella aerogenes* on non-nutrient agar. Northern blots were performed as previously described (Aspegren et al. 2004; Avesson et al. 2011). Briefly, 10 µg total RNA was separated on 8% PAGE/7 M Urea and electroblotted to Hybond N+ nylon membranes (GE Healthcare). After UV cross-linking, immobilized RNA was hybridized with ^32^P-labeled oligonucleotides in Church buffer overnight at 42°C. Signals were analyzed with a Personal Molecular Imager (Bio-Rad) normally after a few hours’ exposure. Membranes analyzed more than once were stripped with 0.1 × SSC/1% SDS buffer for 1 h at 95°C and controlled for residual signal before reprobing. Oligonucleotide sequences are provided in Supplemental Table S6.

### RNA-seq validation

Strain growth and RNA extraction for RNA-seq have been described previously (Glöckner et al. 2016). For each strain, RNA was prepared from growing cells and two multicellular developmental stages: aggregates and tipped aggregates/fingers (biological duplicates except for *D. lacteum*). TruSeq small RNA Sample Preparation kit (Illumina RS- 200-0012) was used to prepare sequencing libraries from 1 µg total RNA. The library preparation was performed according to the manufacturer's protocol (#15004197 rev G) in which cDNA representing 18–70 nt RNA were isolated. Single-read 50-bp sequencing was performed using v4 sequencing chemistry on an Illumina HiSeq 2500. To reduce influence of degradation products, only full-length 50-bp reads were mapped with Bowtie allowing for one mismatch (Langmead et al. 2009) and counted with featureCounts (Liao et al. 2014). Read coverage over Class I candidate loci were calculated with BEDTools genomecov v. 2.26.0 (Quinlan and Hall 2010). Class I RNAs were considered to be validated by RNA-seq if the read coverage indicated a distinct 5′ end and reads specifically matched the predicted loci, that is, did not appear to be part of a considerably longer transcript. Principal component analyses were performed using DESeq2 (Love et al. 2014). ROC plot evaluation of Class I prediction was performed using pROC package (Robin et al. 2011) in R (R Core Team 2017).

### Strains and genomic resources

Strain names and accession numbers for genomic sequences are listed in Supplemental Table S1.

### Identification and analyses of Class I RNAs

Of the 40 *D. discoideum* Class I RNAs annotated previous to this study, six were excluded from the model build (r48, r53, r54, r55, r58, and r61) because they represented truncated fragments or lacked the canonical features such as the stem or conserved sequence motif. The remaining 34 were aligned with MAFFT v7.407 (Katoh and Standley 2013) using the ginsi setting. Consensus structure for the alignment was predicted with RNAalifold 2.3.3 (Bernhart et al. 2008) using the -T 22 option to account for the optimal growing temperature of the amoebas. Alignment and consensus structure was combined to a Stockholm alignment file, and a covariance model was created with Infernal 1.1.2 (Nawrocki and Eddy 2013). Infernal was then used to search the genomes of *D. discoideum*, *D. purpureum*, *D. lacteum*, *P. pallidum*, *A. subglobosum,* and *D. fasciculatum* using default settings, and candidates with a score ≥ 25 were added to the alignment using MAFFT (ginsi –add). The new alignment was manually curated and used to predict consensus structure, create a new covariance model, and perform a new search in the same genomes. This procedure was iterated six times, that is, until no new candidates with an Infernal score ≥ 25 were identified. Enriched sequence motifs were identified up to 150 nt upstream of identified candidates with MEME v. 5.0.3 (Bailey and Elkan 1994). For final Class I identification, Infernal searches were performed with increased sensitivity, and all candidates scoring 15 or higher were kept (cmsearch –nohmm –notrunc -T 15). The candidates were then evaluated based on the presence of DUSE and TGTG box in the 150 nt preceding the predicted start of transcription (see above) using FIMO v. 5.0.3 (Grant et al. 2011). Infernal score, FIMO motif score, and a motif distance score (+5) were then added to a total score. Missing DUSE or incorrect distance was penalized with −10 or −5, respectively (Supplemental Code). If a total score of 32 was achieved, the candidate was considered likely to be an expressed true Class I RNA and kept for further analyses. Representative sequence logos of manually curated sequence alignments (mafft ‐‐maxiterate 1000 –localpair) were created with WebLogo 3 (Crooks 2004). For each Class I RNA, a maximum expected accuracy secondary structure was predicted with RNAfold (rnafold -T 22 –MEA). Based on this, the percentages of predicted base-pairing were calculated and plotted for both the entire Class I RNA as well as the 11-nt motif region (Supplemental Code). Class I RNA GC content, lengths, and genomic distribution were analyzed and plotted with in-house Python scripts (Supplemental Code).

### Ortholog identification and shared synteny search

For Dictyostelia species lacking genome annotations, gene prediction was performed with Gene id v. 1.4 (Blanco et al. 2007) using the Dictyostelium parameter file. Ortholog identification was performed using OrthoFinder v. 2.3.3 (Emms and Kelly 2015). Protein domain architectures for *rnp1A* orthologs were analyzed with hmmscan using the HMMER web interface (Potter et al. 2018). Ortholog information for *D. discoideum*, *D. firmibasis*, *D. lacteum*, *P. pallidum*, *A. subglobosum,* and *D. fasciculatum* was used to investigate shared synteny for Class I RNA loci. For each Class I RNA locus, gene information within a 10-kb flanking region was retrieved. Next, we searched for orthologs of these genes in the other organisms included in the search. If a Class I RNA was found within 10 kb of an orthologous gene in another organism, the genomic context was drawn (Supplemental Code) and manually inspected to determine the level of shared synteny.

## Data access

The RNA-seq data generated in this study have been submitted to the NCBI BioProject database (https://www.ncbi.nlm.nih.gov/bioproject/) under accession number PRJNA638268. Python scripts necessary to reproduce analyses and plots are available at GitHub (https://github.com/kjellinjonas/class_I_evo) and in Supplemental Code together with example files. An alignment of RNAs identified in this study have been submitted to Rfam for an update of the Class I RNA family (https://rfam.org/family/RF01414).

## Competing interest statement

The authors declare no competing interests.

## Supplementary Material

Supplemental Material

## References

[GR272856KJEC1] Antolović V, Lenn T, Miermont A, Chubb JR. 2019. Transition state dynamics during a stochastic fate choice. Development 146: dev173740. 10.1242/dev.173740 30890571 PMC6602359

[GR272856KJEC2] Aspegren A, Hinas A, Larsson P, Larsson A, Söderbom F. 2004. Novel non-coding RNAs in *Dictyostelium discoideum* and their expression during development. Nucleic Acids Res 32: 4646–4656. 10.1093/nar/gkh804 15333696 PMC516072

[GR272856KJEC3] Avesson L, Schumacher HT, Fechter P, Romby P, Hellman U, Söderbom F. 2011. Abundant class of non-coding RNA regulates development in the social amoeba *Dictyostelium discoideum*. RNA Biol 8: 1094–1104. 10.4161/rna.8.6.17214 21941123

[GR272856KJEC4] Avesson L, Reimegård J, Wagner EGH, Söderbom F. 2012. MicroRNAs in Amoebozoa: deep sequencing of the small RNA population in the social amoeba *Dictyostelium discoideum* reveals developmentally regulated microRNAs. RNA 18: 1771–1782. 10.1261/rna.033175.112 22875808 PMC3446702

[GR272856KJEC5] Bailey TL, Elkan C. 1994. Fitting a mixture model by expectation maximization to discover motifs in biopolymers. Proc Int Conf Intell Syst Mol Biol 2: 28–36. 7584402

[GR272856KJEC6] Bernhart SH, Hofacker IL, Will S, Gruber AR, Stadler PF. 2008. RNAalifold: improved consensus structure prediction for RNA alignments. BMC Bioinformatics 9: 474. 10.1186/1471-2105-9-474 19014431 PMC2621365

[GR272856KJEC7] Blanco E, Parra G, Guigó R. 2007. Using geneid to identify genes. Curr Protoc Bioinformatics Chapter 4: Unit 4.3. 10.1002/0471250953.bi0403s18 18428791

[GR272856KJEC8] Brown MW, Spiegel FW, Silberman JD. 2009. Phylogeny of the “forgotten” cellular slime mold, *Fonticula alba*, reveals a key evolutionary branch within Opisthokonta. Mol Biol Evol 26: 2699–2709. 10.1093/molbev/msp185 19692665

[GR272856KJEC9] Brown MW, Silberman JD, Spiegel FW. 2011. “Slime molds” among the Tubulinea (Amoebozoa): molecular systematics and taxonomy of *Copromyxa*. Protist 162: 277–287. 10.1016/j.protis.2010.09.003 21112814

[GR272856KJEC10] Brown MW, Kolisko M, Silberman JD, Roger AJ. 2012a. Aggregative multicellularity evolved independently in the eukaryotic supergroup Rhizaria. Curr Biol 22: 1123–1127. 10.1016/j.cub.2012.04.021 22608512

[GR272856KJEC11] Brown MW, Silberman JD, Spiegel FW. 2012b. A contemporary evaluation of the acrasids (Acrasidae, Heterolobosea, Excavata). Eur J Protistol 48: 103–123. 10.1016/j.ejop.2011.10.001 22154141

[GR272856KJEC12] Burki F, Roger AJ, Brown MW, Simpson AGB. 2020. The new tree of eukaryotes. Trends Ecol Evol 35: 43–55. 10.1016/j.tree.2019.08.008 31606140

[GR272856KJEC13] Cech TR, Steitz JA. 2014. The noncoding RNA revolution—trashing old rules to forge new ones. Cell 157: 77–94. 10.1016/j.cell.2014.03.008 24679528

[GR272856KJEC14] Crooks GE. 2004. Weblogo: a sequence logo generator. Genome Res 14: 1188–1190. 10.1101/gr.849004 15173120 PMC419797

[GR272856KJEC15] Deline B, Greenwood JM, Clark JW, Puttick MN, Peterson KJ, Donoghue PCJ. 2018. Evolution of metazoan morphological disparity. Proc Natl Acad Sci 115: E8909–E8918. 10.1073/pnas.1810575115 30181261 PMC6156614

[GR272856KJEC16] dos Reis M, Thawornwattana Y, Angelis K, Telford MJ, Donoghue PCJ, Yang Z. 2015. Uncertainty in the timing of origin of animals and the limits of precision in molecular timescales. Curr Biol 25: 2939–2950. 10.1016/j.cub.2015.09.066 26603774 PMC4651906

[GR272856KJEC17] Eichinger L, Pachebat JA, Glöckner G, Rajandream MA, Sucgang R, Berriman M, Song J, Olsen R, Szafranski K, Xu Q, 2005. The genome of the social amoeba *Dictyostelium discoideum*. Nature 435: 43–57. 10.1038/nature03481 15875012 PMC1352341

[GR272856KJEC18] Emms DM, Kelly S. 2015. Orthofinder: solving fundamental biases in whole genome comparisons dramatically improves orthogroup inference accuracy. Genome Biol 16: 157. 10.1186/s13059-015-0721-2 26243257 PMC4531804

[GR272856KJEC19] Gaiti F, Calcino AD, Tanurdžić M, Degnan BM. 2017. Origin and evolution of the metazoan non-coding regulatory genome. Dev Biol 427: 193–202. 10.1016/j.ydbio.2016.11.013 27880868

[GR272856KJEC20] Gaunt SJ, Paul YL. 2012. Changes in *cis*-regulatory elements during morphological evolution. Biology 1: 557–574. 10.3390/biology1030557 24832508 PMC4009813

[GR272856KJEC21] Glöckner G, Lawal HM, Felder M, Singh R, Singer G, Weijer CJ, Schaap P. 2016. The multicellularity genes of dictyostelid social amoebas. Nat Commun 7: 12085. 10.1038/ncomms12085 27357338 PMC4931340

[GR272856KJEC22] Grant CE, Bailey TL, Noble WS. 2011. FIMO: scanning for occurrences of a given motif. Bioinformatics 27: 1017–1018. 10.1093/bioinformatics/btr064 21330290 PMC3065696

[GR272856KJEC23] He D, Fiz-Palacios O, Fu CJ, Fehling J, Tsai CC, Baldauf SL. 2014. An alternative root for the eukaryote tree of life. Curr Biol 24: 465–470. 10.1016/j.cub.2014.01.036 24508168

[GR272856KJEC24] Heidel AJ, Lawal HM, Felder M, Schilde C, Helps NR, Tunggal B, Rivero F, John U, Schleicher M, Eichinger L, 2011. Phylogeny-wide analysis of social amoeba genomes highlights ancient origins for complex intercellular communication. Genome Res 21: 1882–1891. 10.1101/gr.121137.111 21757610 PMC3205573

[GR272856KJEC25] Hildebrandt M, Nellen W. 1992. Differential antisense transcription from the Dictyostelium *EB4* gene locus: implications on antisense-mediated regulation of mRNA stability. Cell 69: 197–204. 10.1016/0092-8674(92)90130-5 1555240

[GR272856KJEC26] Hillmann F, Forbes G, Novohradská S, Ferling I, Riege K, Groth M, Westermann M, Marz M, Spaller T, Winckler T, 2018. Multiple roots of fruiting body formation in Amoebozoa. Genome Biol Evol 10: 591–606. 10.1093/gbe/evy011 29378020 PMC5804921

[GR272856KJEC27] Hinas A, Söderbom F. 2007. Treasure hunt in an amoeba: non-coding RNAs in *Dictyostelium discoideum*. Curr Genet 51: 141–159. 10.1007/s00294-006-0112-z 17171561

[GR272856KJEC28] Hinas A, Larsson P, Avesson L, Kirsebom LA, Virtanen A, Söderbom F. 2006. Identification of the major spliceosomal RNAs in *Dictyostelium discoideum* reveals developmentally regulated U2 variants and polyadenylated snRNAs. Eukaryotic Cell 5: 924–934. 10.1128/EC.00065-06 16757740 PMC1489274

[GR272856KJEC29] Hinas A, Reimegård J, Wagner EGH, Nellen W, Ambros VR, Söderbom F. 2007. The small RNA repertoire of *Dictyostelium discoideum* and its regulation by components of the RNAi pathway. Nucleic Acids Res 35: 6714–6726. 10.1093/nar/gkm707 17916577 PMC2175303

[GR272856KJEC30] Katoh K, Standley DM. 2013. MAFFT multiple sequence alignment software version 7: improvements in performance and usability. Mol Biol Evol 30: 772–780. 10.1093/molbev/mst010 23329690 PMC3603318

[GR272856KJEC31] Kawabe Y, Du Q, Schilde C, Schaap P. 2019. Evolution of multicellularity in Dictyostelia. Int J Dev Biol 63: 359–369. 10.1387/ijdb.190108ps 31840775 PMC6978153

[GR272856KJEC32] Langmead B, Trapnell C, Pop M, Salzberg SL. 2009. Ultrafast and memory-efficient alignment of short DNA sequences to the human genome. Genome Biol 10: R25. 10.1186/gb-2009-10-3-r25 19261174 PMC2690996

[GR272856KJEC33] Lasek-Nesselquist E, Katz LA. 2001. Phylogenetic position of *Sorogena stoianovitchae* and relationships within the class Colpodea (Ciliophora) based on SSU rDNA sequences. J Eukaryot Microbiol 48: 604–607. 10.1111/j.1550-7408.2001.tb00197.x 11596926

[GR272856KJEC34] Liao Y, Smyth GK, Shi W. 2014. Featurecounts: an efficient general purpose program for assigning sequence reads to genomic features. Bioinformatics 30: 923–930. 10.1093/bioinformatics/btt656 24227677

[GR272856KJEC35] Liao Z, Kjellin J, Hoeppner MP, Grabherr M, Söderbom F. 2018. Global characterization of the Dicer-like protein DrnB roles in miRNA biogenesis in the social amoeba *Dictyostelium discoideum*. RNA Biol 15: 937–954. 10.1080/15476286.2018.1481697 29966484 PMC6161686

[GR272856KJEC36] Love MI, Huber W, Anders S. 2014. Moderated estimation of fold change and dispersion for RNA-seq data with DESeq2. Genome Biol 15: 550. 10.1186/s13059-014-0550-8 25516281 PMC4302049

[GR272856KJEC37] Meier D, Kruse J, Buttlar J, Friedrich M, Zenk F, Boesler B, Förstner KU, Hammann C, Nellen W. 2016. Analysis of the microprocessor in Dictyostelium: the role of RbdB, a dsRNA binding protein. PLoS Genet 12: e1006057. 10.1371/journal.pgen.1006057 27272207 PMC4894637

[GR272856KJEC38] Mosig A, Sameith K, Stadler P. 2006. Fragrep: an efficient search tool for fragmented patterns in genomic sequences. Genomics Proteomics Bioinformatics 4: 56–60. 10.1016/S1672-0229(06)60017-X 16689703 PMC5054030

[GR272856KJEC39] Muñoz-Dorado J, Marcos-Torres FJ, García-Bravo E, Moraleda-Muñoz A, Pérez J. 2016. Myxobacteria: moving, killing, feeding, and surviving together. Front Microbiol 7: 781. 10.3389/fmicb.2016.00781 27303375 PMC4880591

[GR272856KJEC40] Nawrocki EP, Eddy SR. 2013. Infernal 1.1: 100-fold faster RNA homology searches. Bioinformatics 29: 2933–2935. 10.1093/bioinformatics/btt509 24008419 PMC3810854

[GR272856KJEC41] Ngo T, Miao X, Robinson DN, Zhou Q. 2016. An RNA-binding protein, RNP-1, protects microtubules from nocodazole and localizes to the leading edge during cytokinesis and cell migration in Dictyostelium cells. Acta Pharmacol Sin 37: 1449–1457. 10.1038/aps.2016.57 27569394 PMC5099410

[GR272856KJEC42] Parikh A, Miranda ER, Katoh-Kurasawa M, Fuller D, Rot G, Zagar L, Curk T, Sucgang R, Chen R, Zupan B, 2010. Conserved developmental transcriptomes in evolutionarily divergent species. Genome Biol 11: R35. 10.1186/gb-2010-11-3-r35 20236529 PMC2864575

[GR272856KJEC43] Potter SC, Luciani A, Eddy SR, Park Y, Lopez R, Finn RD. 2018. HMMER web server: 2018 update. Nucleic Acids Res 46: W200–W204. 10.1093/nar/gky448 29905871 PMC6030962

[GR272856KJEC44] Quinlan AR, Hall IM. 2010. BEDTools: a flexible suite of utilities for comparing genomic features. Bioinformatics 26: 841–842. 10.1093/bioinformatics/btq033 20110278 PMC2832824

[GR272856KJEC45] R Core Team. 2017. R: a language and environment for statistical computing. R Foundation for Statistical Computing, Vienna. https://www.R-project.org/.

[GR272856KJEC46] Richard P, Manley JL. 2009. Transcription termination by nuclear RNA polymerases. Genes Dev 23: 1247–1269. 10.1101/gad.1792809 19487567 PMC2763537

[GR272856KJEC47] Robin X, Turck N, Hainard A, Tiberti N, Lisacek F, Sanchez JC, Müller M. 2011. pROC: an open-source package for R and S+ to analyze and compare ROC curves. BMC Bioinformatics 12: 77. 10.1186/1471-2105-12-77 21414208 PMC3068975

[GR272856KJEC48] Romeralo M, Skiba A, Gonzalez-Voyer A, Schilde C, Lawal H, Kedziora S, Cavender JC, Glöckner G, Urushihara H, Schaap P. 2013. Analysis of phenotypic evolution in Dictyostelia highlights developmental plasticity as a likely consequence of colonial multicellularity. Proc R Soc B: Biol Sci 280: 20130976. 10.1098/rspb.2013.0976 PMC3712420 23782883

[GR272856KJEC49] Rosengarten RD, Santhanam B, Fuller D, Katoh-Kurasawa M, Loomis WF, Zupan B, Shaulsky G. 2015. Leaps and lulls in the developmental transcriptome of *Dictyostelium discoideum*. BMC Genomics 16: 294. 10.1186/s12864-015-1491-7 25887420 PMC4403905

[GR272856KJEC50] Rosengarten RD, Santhanam B, Kokosar J, Shaulsky G. 2017. The long noncoding RNA transcriptome of *Dictyostelium discoideum* development. G3 (Bethesda) 7: 387–398. 10.1534/g3.116.037150 27932387 PMC5295588

[GR272856KJEC51] Schilde C, Skiba A, Schaap P. 2014. Evolutionary reconstruction of pattern formation in 98 Dictyostelium species reveals that cell-type specialization by lateral inhibition is a derived trait. Evodevo 5: 34. 10.1186/2041-9139-5-34 25904998 PMC4406040

[GR272856KJEC52] Schilde C, Lawal HM, Kin K, Shibano-Hayakawa I, Inouye K, Schaap P. 2019. A well supported multi gene phylogeny of 52 dictyostelia. Mol Phylogenet Evol 134: 66–73. 10.1016/j.ympev.2019.01.017 30711536 PMC6430600

[GR272856KJEC53] Sheikh S, Thulin M, Cavender JC, Escalante R, Kawakami SI, Lado C, Landolt JC, Nanjundiah V, Queller DC, Strassmann JE, 2018. A new classification of the dictyostelids. Protist 169: 1–28. 10.1016/j.protis.2017.11.001 29367151

[GR272856KJEC54] Sucgang R, Kuo A, Tian X, Salerno W, Parikh A, Feasley CL, Dalin E, Tu H, Huang E, Barry K, 2011. Comparative genomics of the social amoebae *Dictyostelium discoideum* and *Dictyostelium purpureum*. Genome Biol 12: R20. 10.1186/gb-2011-12-2-r20 21356102 PMC3188802

[GR272856KJEC55] Tice AK, Silberman JD, Walthall AC, Le KND, Spiegel FW, Brown MW. 2016. *Sorodiplophrys stercorea*: another novel lineage of sorocarpic multicellularity. J Eukaryot Microbiol 63: 623–628. 10.1111/jeu.12311 26940948

[GR272856KJEC56] Urushihara H, Kuwayama H, Fukuhara K, Itoh T, Kagoshima H, Shin-I T, Toyoda A, Ohishi K, Taniguchi T, Noguchi H, 2015. Comparative genome and transcriptome analyses of the social amoeba *Acytostelium subglobosum* that accomplishes multicellular development without germ-soma differentiation. BMC Genomics 16: 80. 10.1186/s12864-015-1278-x 25758444 PMC4334915

