# Supplementary material for: Abundantly expressed class of noncoding RNAs conserved through the multicellular evolution of dictyostelid social amoebas
Source: Genome Res. 2021 Mar;31(3):436–47. doi: 10.1101/gr.272856.120 (PMC7919456; doi:10.1101/gr.272856.120)
Supplement: Supplemental Material [file supp_31_3_436__index.html]

Abundantly expressed class of noncoding RNAs conserved through the multicellular evolution of dictyostelid social amoebas — Abundantly expressed class of noncoding RNAs conserved through the multicellular evolution of dictyostelid social amoebas — Supplemental Material 

# Abundantly expressed class of noncoding RNAs conserved through the multicellular evolution of dictyostelid social amoebas

## Supplemental Material

- Supplemental\_Code.zip
- Supplemental\_Fig\_S7.pdf
- Supplemental\_Fig\_S8.pdf
- Supplemental\_Material.pdf
- Supplemental\_Table\_S1.xlsx
- Supplemental\_Table\_S2.xlsx
- Supplemental\_Table\_S3.xlsx
- Supplemental\_Table\_S4.xlsx
- Supplementary\_Table\_S5.xlsx
