# Supplementary material for: Abundantly expressed class of noncoding RNAs conserved through the multicellular evolution of dictyostelid social amoebas
Source: Genome Res. 2021 Mar;31(3):436–47. doi: 10.1101/gr.272856.120 (PMC7919456; doi:10.1101/gr.272856.120)
Supplement: Supplemental Material [file supp_gr.272856.120_Supplemental_Fig_S7.pdf]

Supplemental Figure S7. Genomic distribution of Class I RNA loci.

Chromosome/contig/scaffold names are indicated to the left and respective lengths are normalized to the longest one (length in bp are presented to the very right of each schematic stretch of DNA). Total number of Class I RNA genes for each organism is given at the top and for each chromosome/contig/scaffold to the very right. Class I RNA genes are indicated by black arrows except for genes with identical sequences, which are indicated with colored arrows. Short vertical lines specify every 0.5 mbp. In *D. discoideum*, the duplication on chromosome 2 (DDB0232429) is indicated by grey boxes with vertical lines.

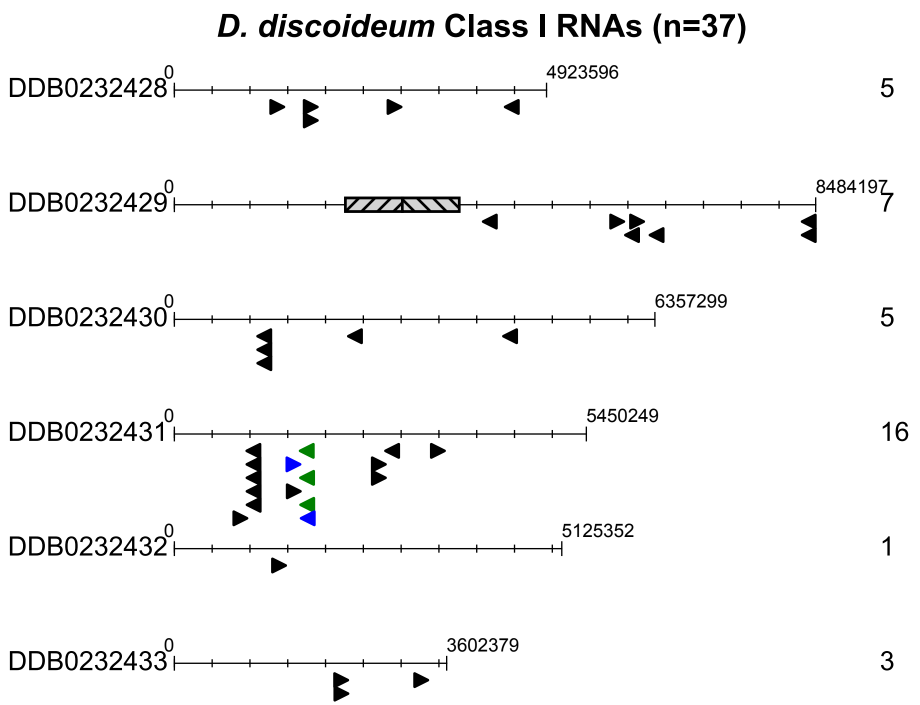
D. discoideum Class I RNAs (n=37)
DDB0232428
0
4923596
5
DDB0232429
0
8484197
7
DDB0232430
0
6357299
5
DDB0232431
0
5450249
16
DDB0232432
0
5125352
1
DDB0232433
0
3602379
3

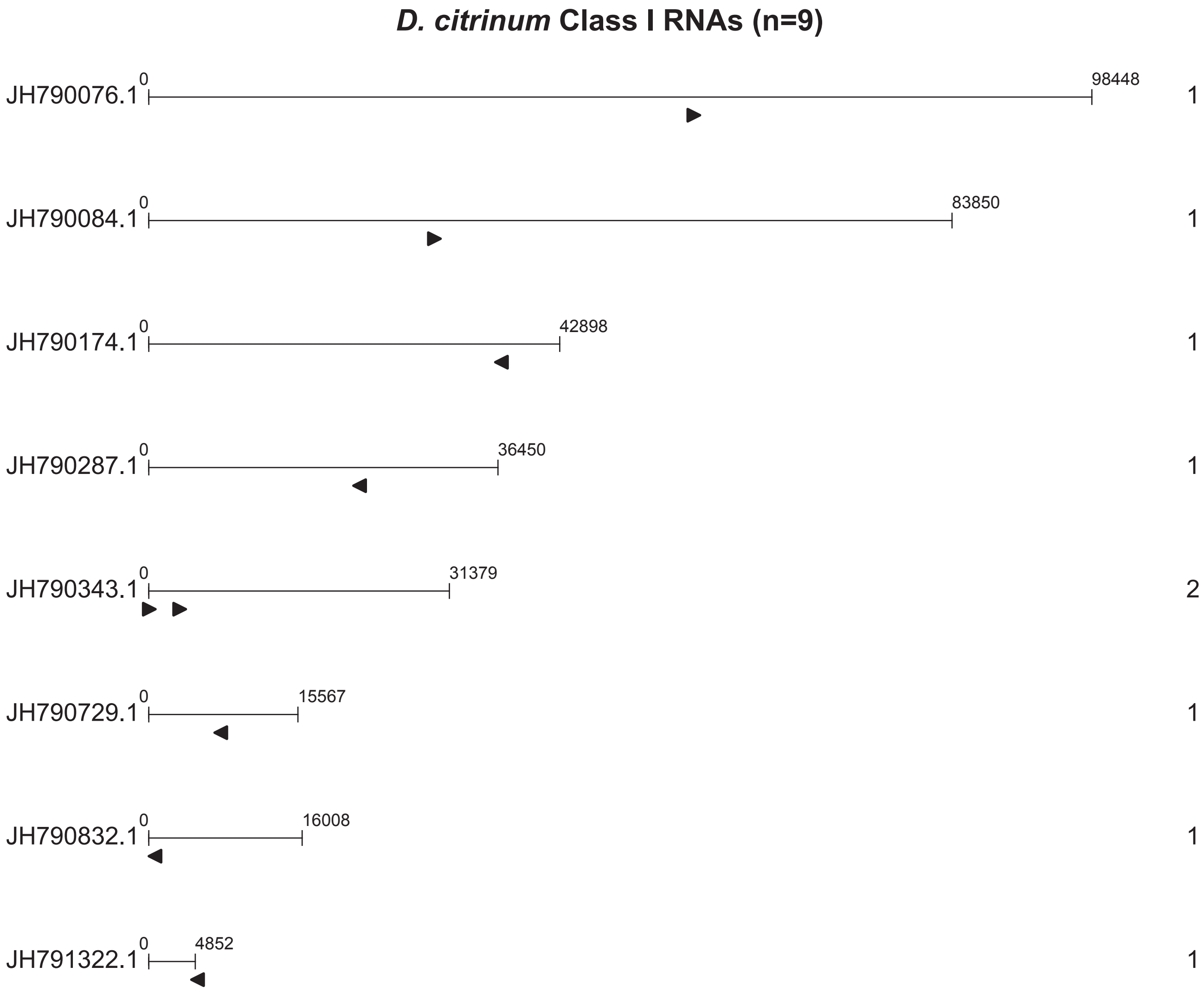
D. citrinum Class I RNAs (n=9)
JH790076.1
0
98448
1
JH790084.1
0
83850
1
JH790174.1
0
42898
1
JH790287.1
0
36450
1
JH790343.1
0
31379
2
JH790729.1
0
15567
1
JH790832.1
0
16008
1
JH791322.1
0
4852
1

D. intermedium Class I RNAs (n=22)

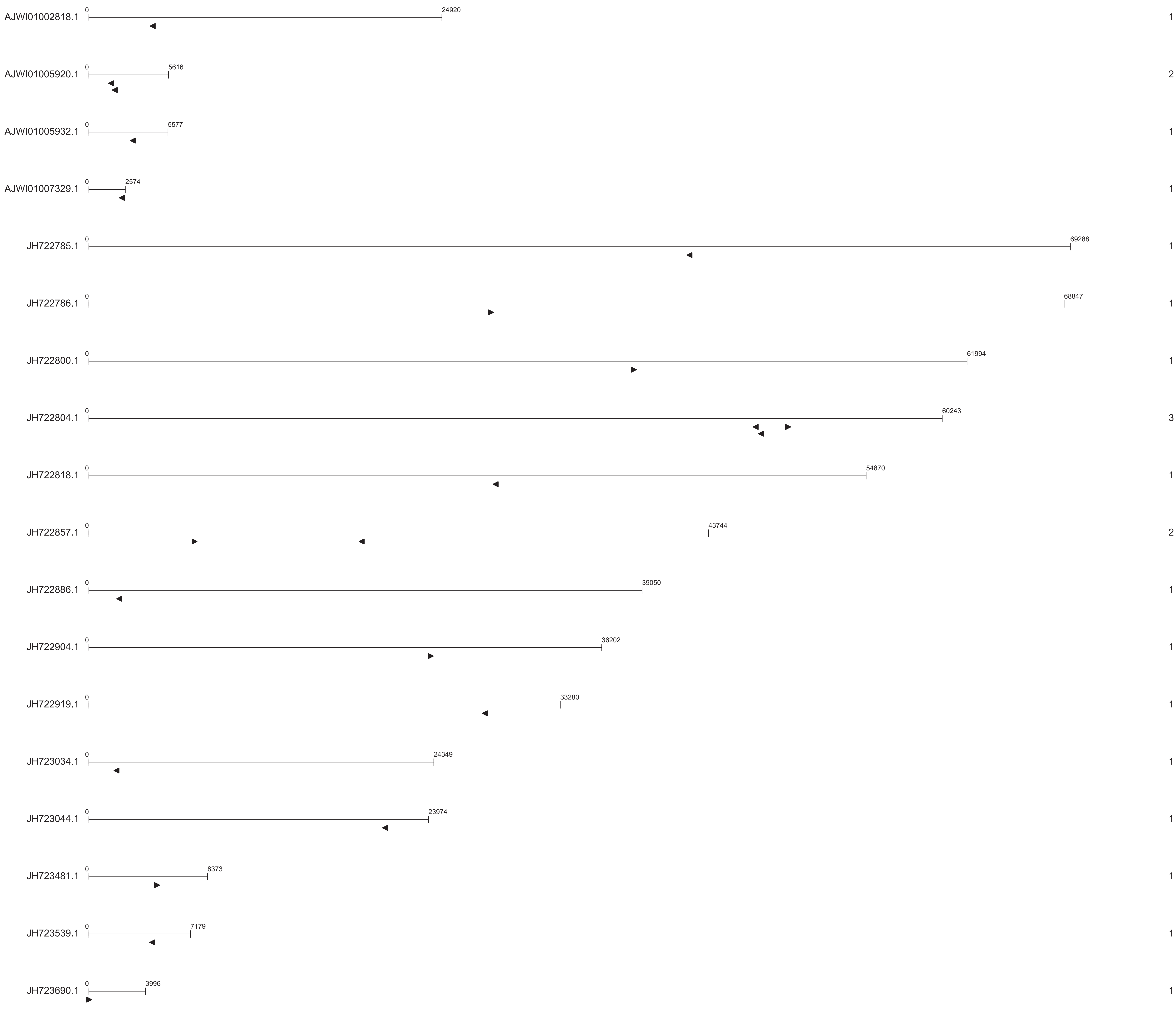

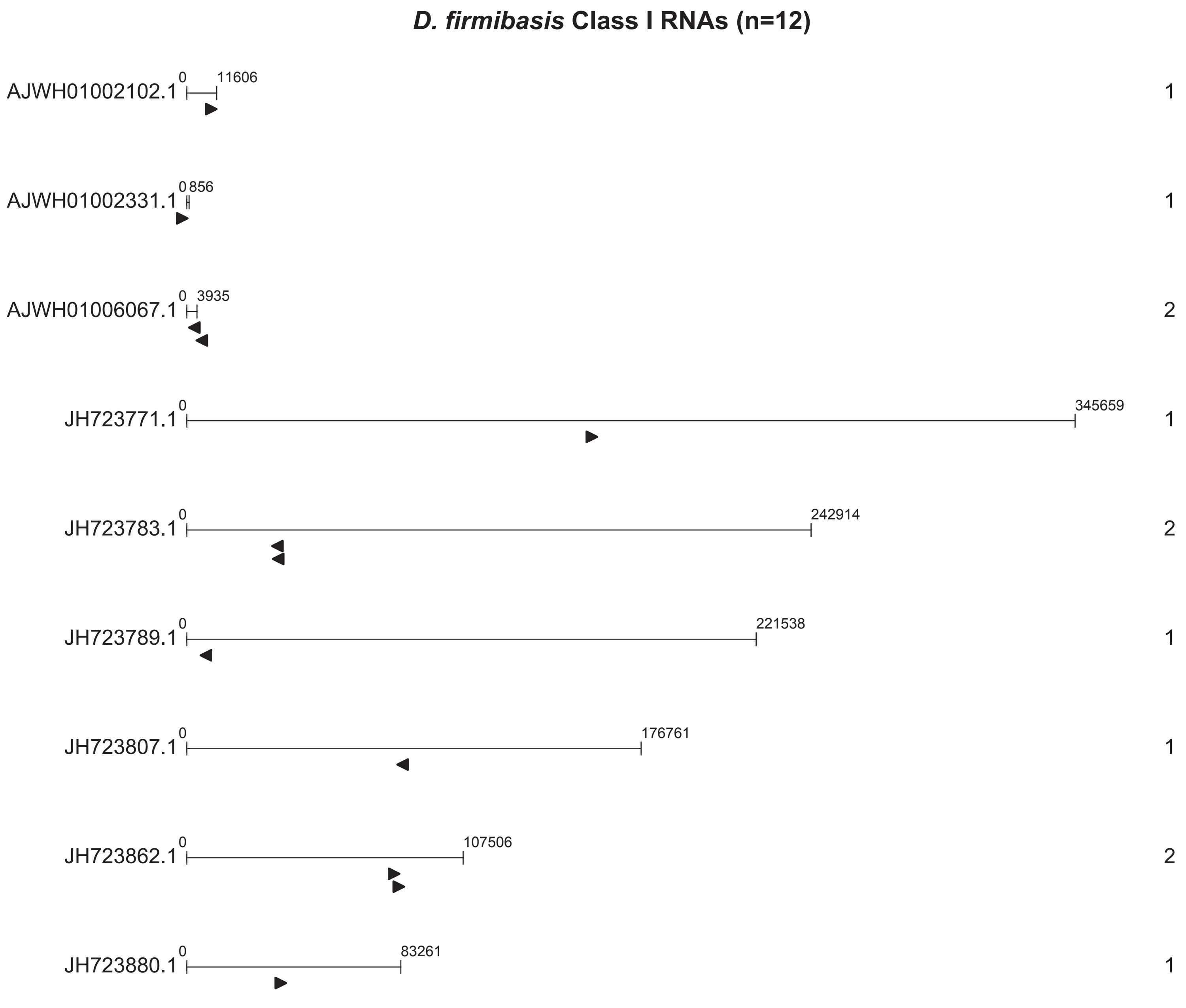
D. firmibasis Class I RNAs (n=12)
AJWH01002102.1
0
11606
1
AJWH01002331.1
0
856
1
AJWH01006067.1
0
3935
2
JH723771.1
0
345659
1
JH723783.1
0
242914
2
JH723789.1
0
221538
1
JH723807.1
0
176761
1
JH723862.1
0
107506
2
JH723880.1
0
83261
1

***D. purpureum* Class I RNAs (n=30)**

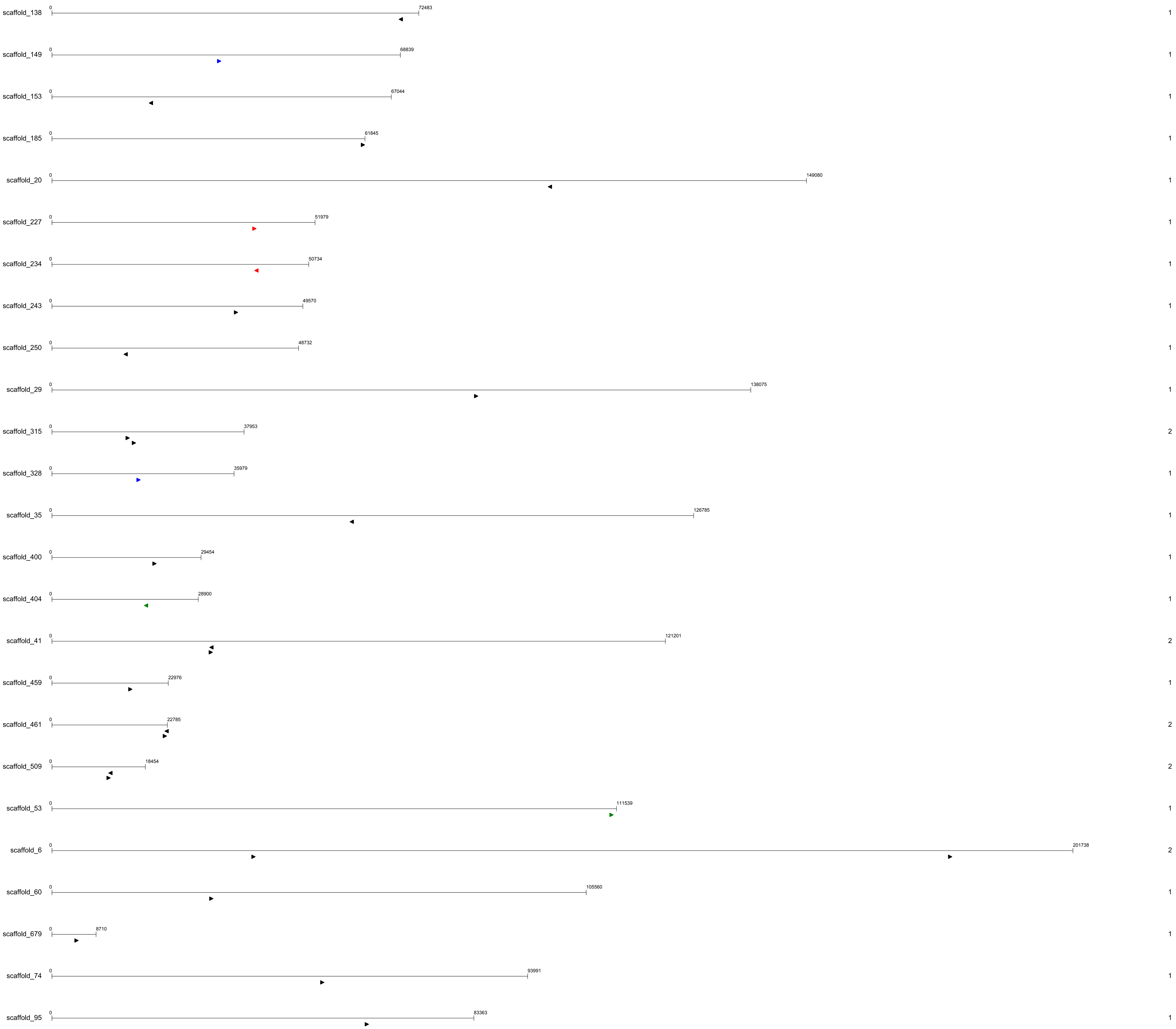

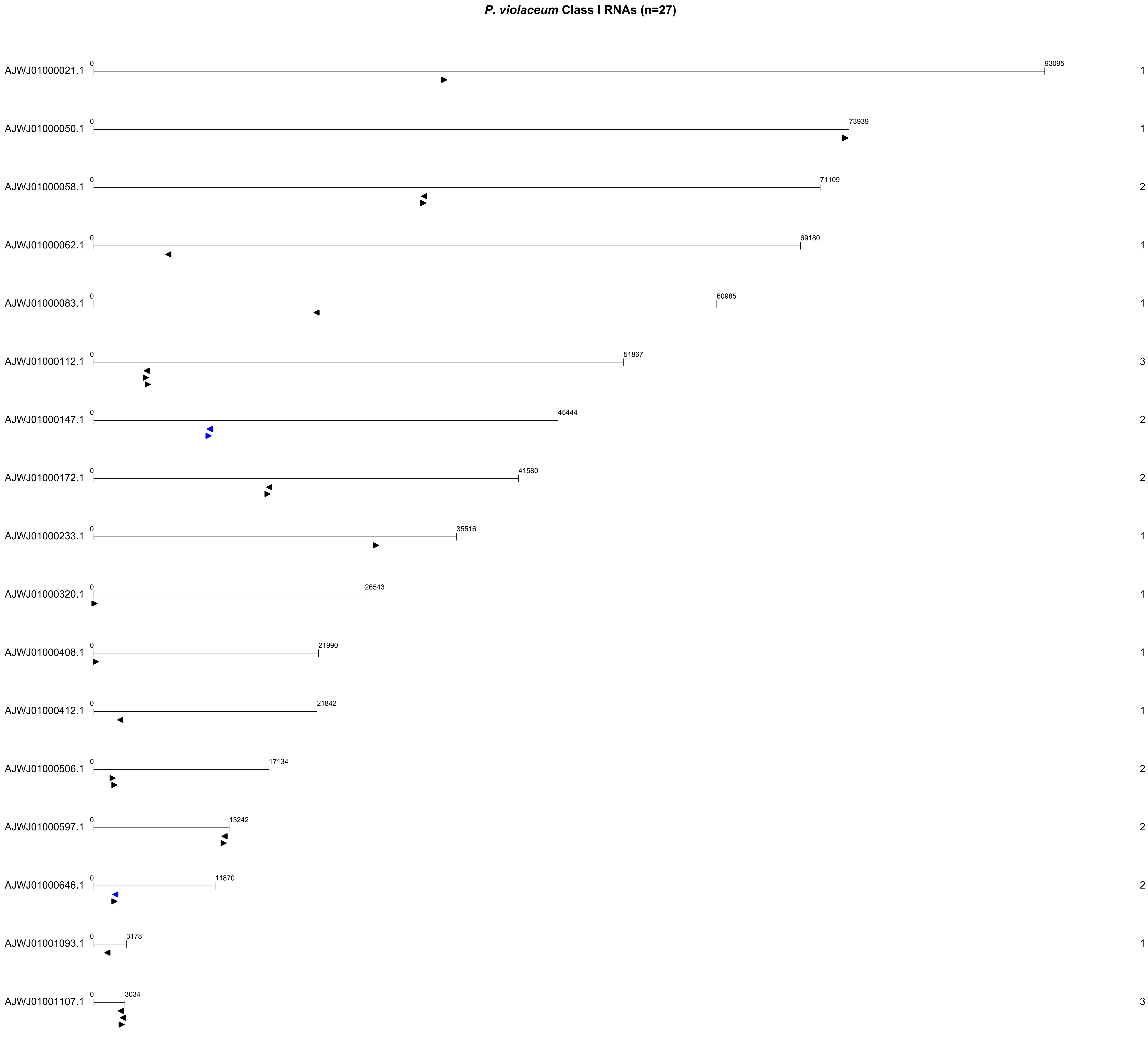

P. violaceum Class I RNAs (n=27)
AJWJ01000021.1
0
93095
1
AJWJ01000050.1
0
73939
1
AJWJ01000058.1
0
71109
2
AJWJ01000062.1
0
69180
1
AJWJ01000083.1
0
60985
1
AJWJ01000112.1
0
51867
3
AJWJ01000147.1
0
45444
2
AJWJ01000172.1
0
41580
2
AJWJ01000233.1
0
35516
1
AJWJ01000320.1
0
26543
1
AJWJ01000408.1
0
21990
1
AJWJ01000412.1
0
21842
1
AJWJ01000506.1
0
17134
2
AJWJ01000597.1
0
13242
2
AJWJ01000646.1
0
11870
2
AJWJ01001093.1
0
3178
1
AJWJ01001107.1
0
3034
3

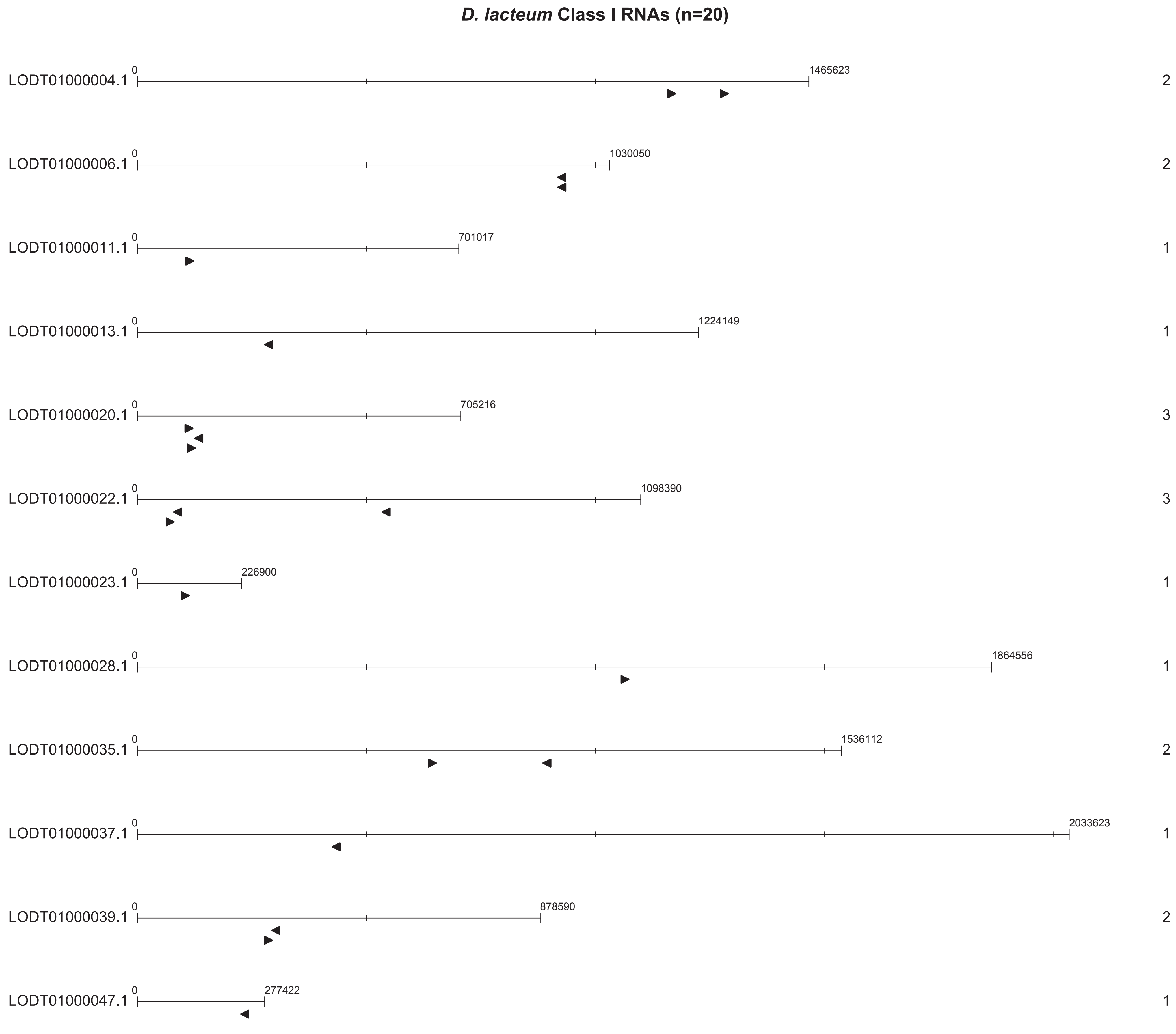
D. lacteum Class I RNAs (n=20)
LODT01000004.1
0
1465623
2
LODT01000006.1
0
1030050
2
LODT01000011.1
0
701017
1
LODT01000013.1
0
1224149
1
LODT01000020.1
0
705216
3
LODT01000022.1
0
1098390
3
LODT01000023.1
0
226900
1
LODT01000028.1
0
1864556
1
LODT01000035.1
0
1536112
2
LODT01000037.1
0
2033623
1
LODT01000039.1
0
878590
2
LODT01000047.1
0
277422
1

***D. caveatum* Class I RNAs (n=24)**

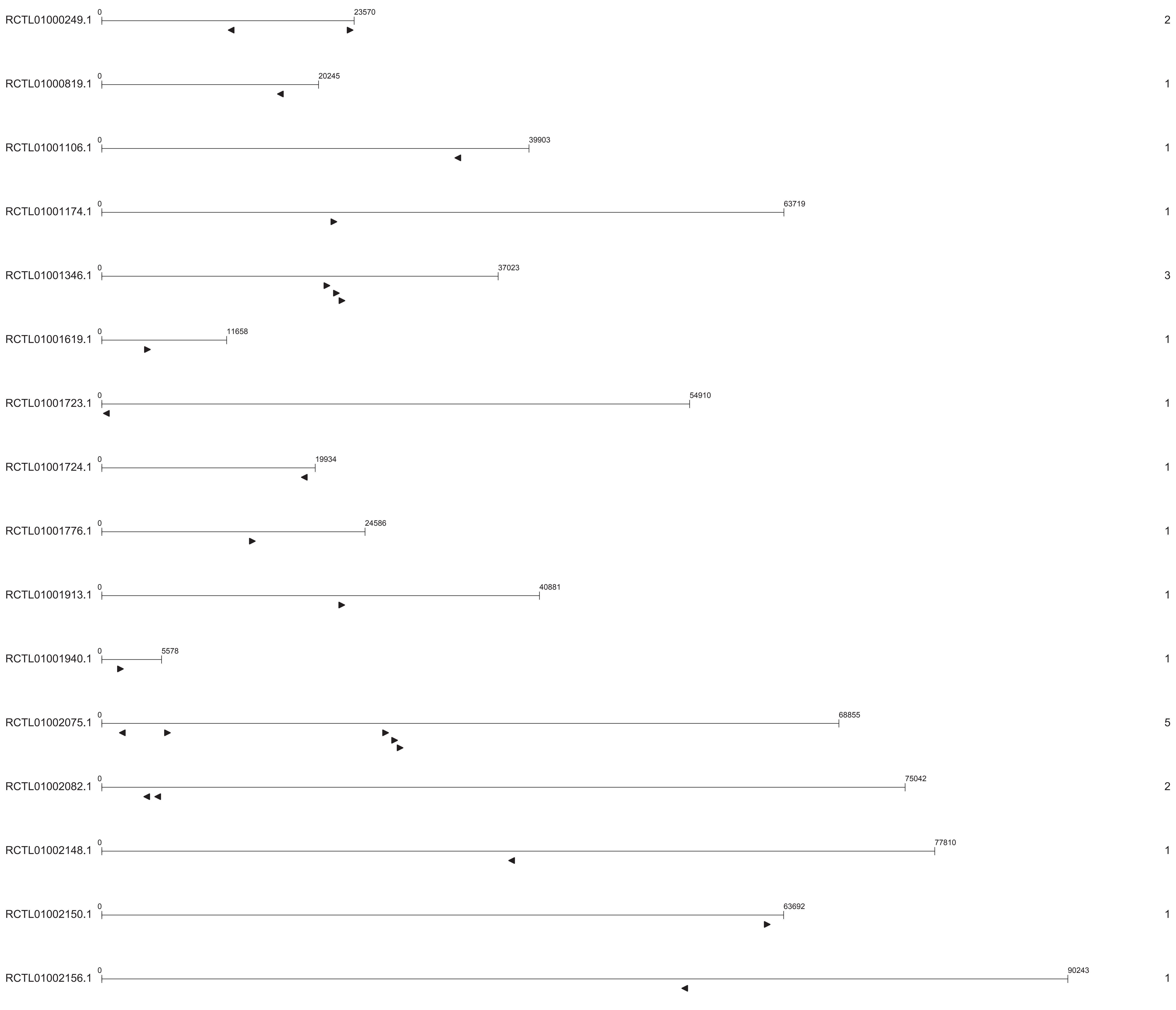

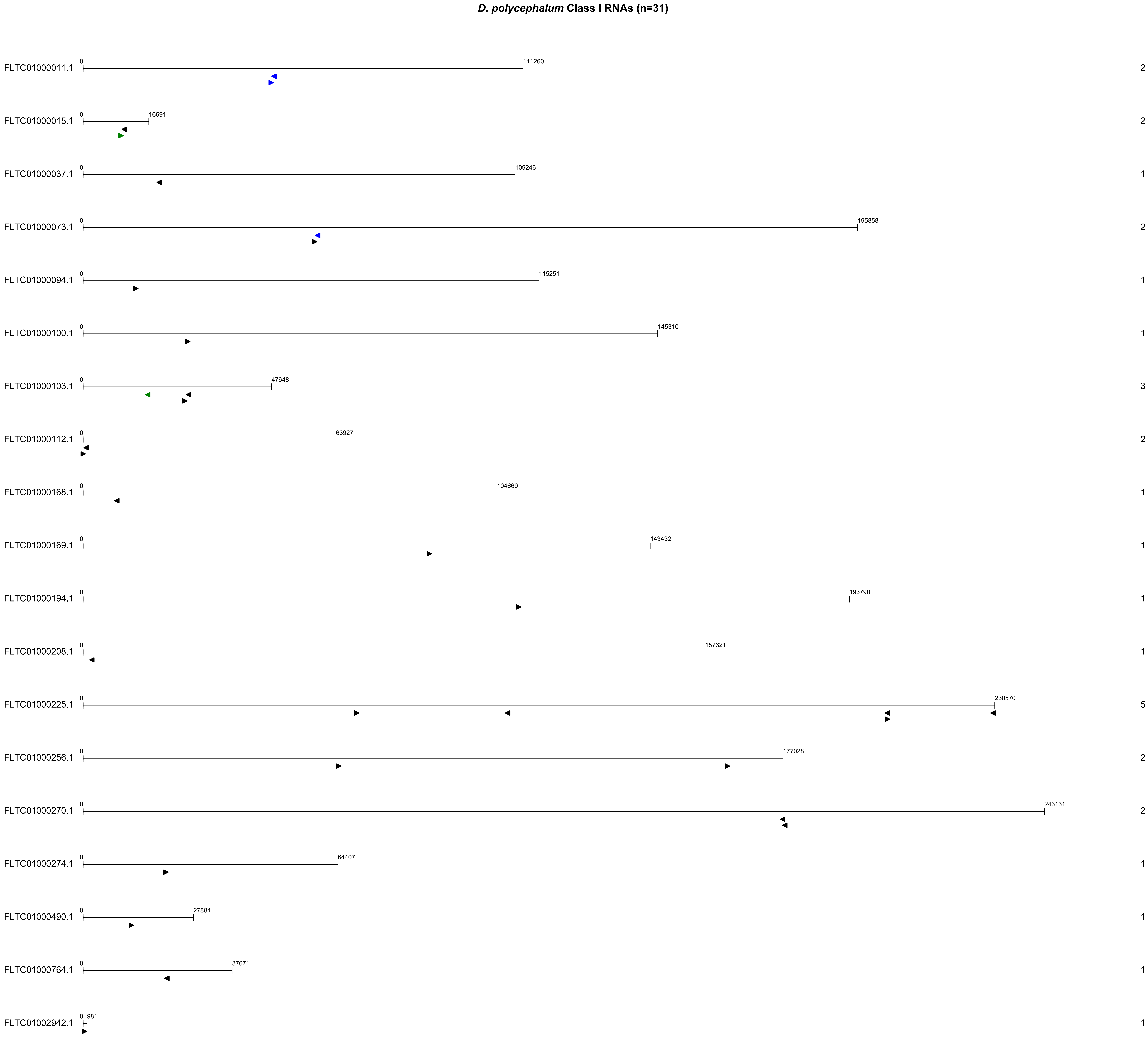

D. polycephalum Class I RNAs (n=31)
FLTC01000011.1
0
111260
FLTC01000015.1
16591
FLTC01000037.1
109246
FLTC01000073.1
195858
FLTC01000094.1
115251
FLTC01000100.1
145310
FLTC01000103.1
47648
FLTC01000112.1
63927
FLTC01000168.1
104669
FLTC01000169.1
143432
FLTC01000194.1
193790
FLTC01000208.1
157321
FLTC01000225.1
230570
FLTC01000256.1
177028
FLTC01000270.1
243131
FLTC01000274.1
64407
FLTC01000490.1
27884
FLTC01000764.1
37671
FLTC01002942.1
981

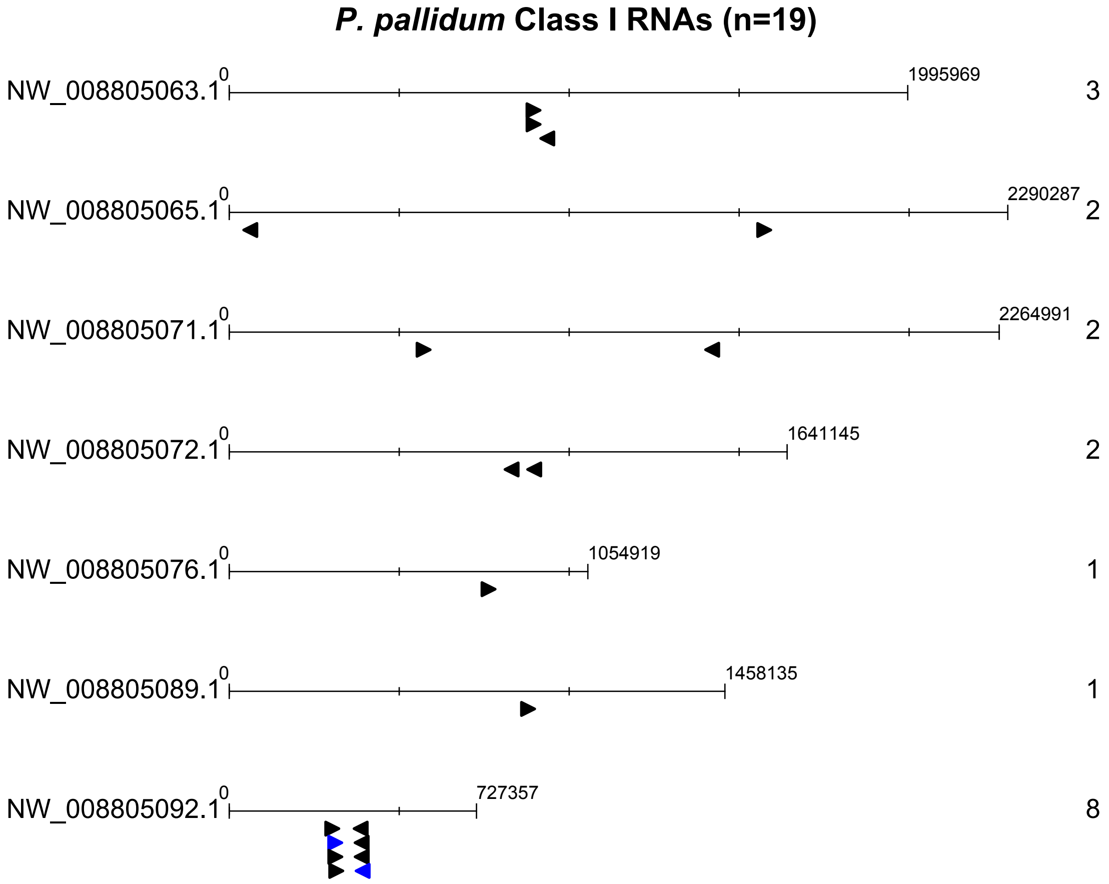
P. pallidum Class I RNAs (n=19)
NW_008805063.1
0
1995969
3
NW_008805065.1
0
2290287
2
NW_008805071.1
0
2264991
2
NW_008805072.1
0
1641145
2
NW_008805076.1
0
1054919
1
NW_008805089.1
0
1458135
1
NW_008805092.1
0
727357
8

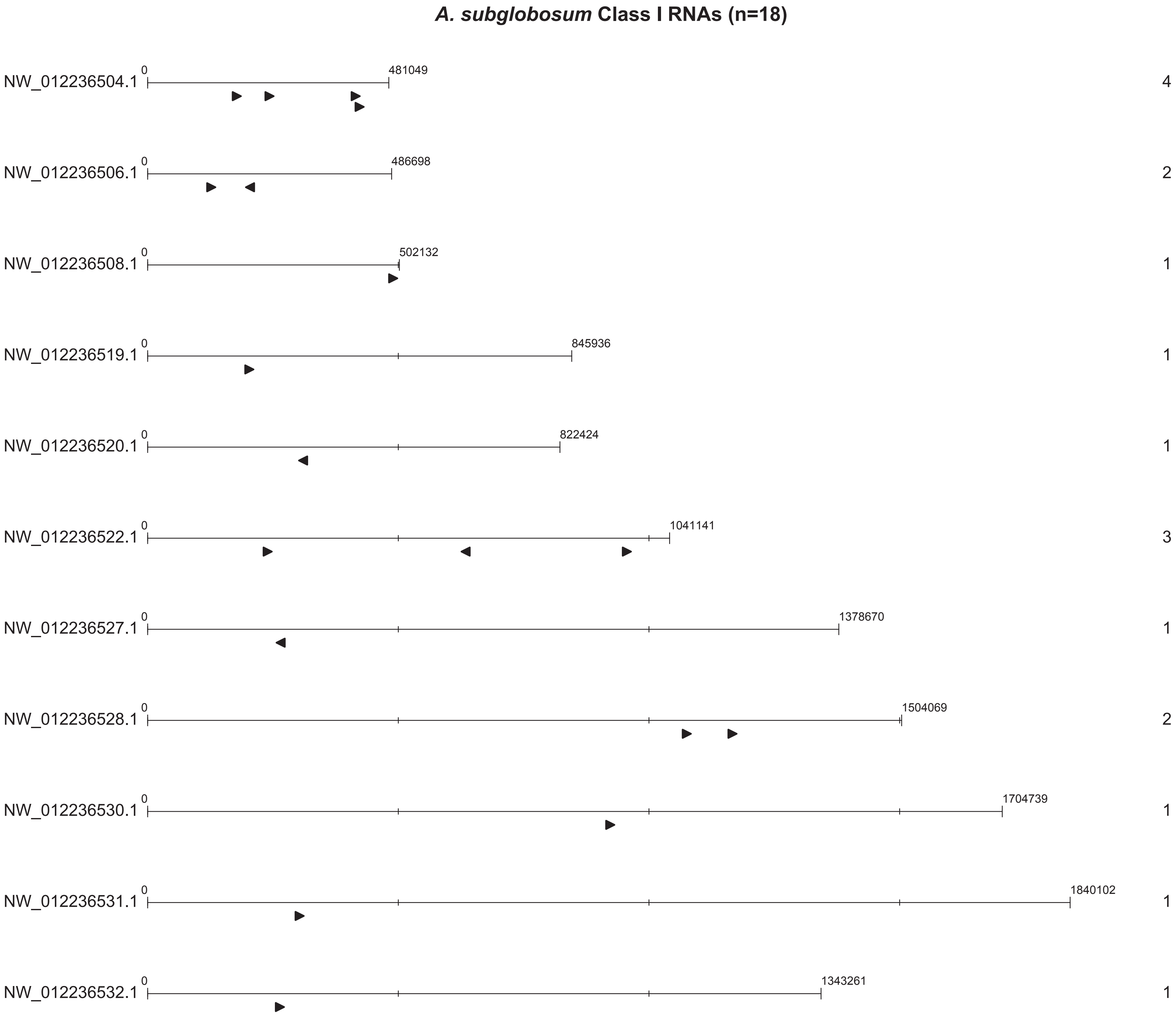
A. subglobosum Class I RNAs (n=18)
NW_012236504.1
0
481049
4
NW_012236506.1
0
486698
2
NW_012236508.1
0
502132
1
NW_012236519.1
0
845936
1
NW_012236520.1
0
822424
1
NW_012236522.1
0
1041141
3
NW_012236527.1
0
1378670
1
NW_012236528.1
0
1504069
2
NW_012236530.1
0
1704739
1
NW_012236531.1
0
1840102
1
NW_012236532.1
0
1343261
1

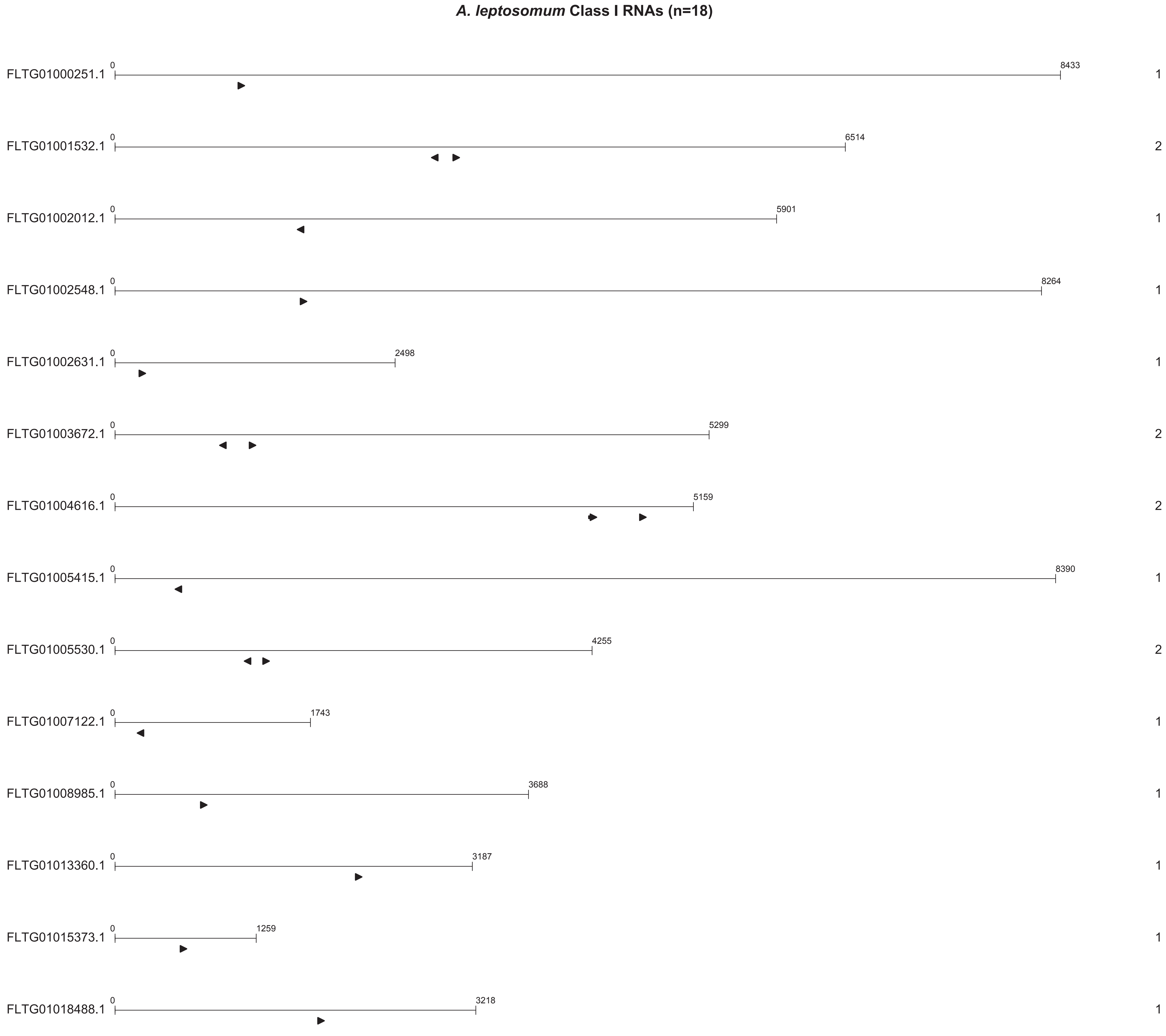

A. leptosomum Class I RNAs (n=18)
FLTG01000251.1
0
8433
1
FLTG01001532.1
0
6514
2
FLTG01002012.1
0
5901
1
FLTG01002548.1
0
8264
1
FLTG01002631.1
0
2498
1
FLTG01003672.1
0
5299
2
FLTG01004616.1
0
5159
2
FLTG01005415.1
0
8390
1
FLTG01005530.1
0
4255
2
FLTG01007122.1
0
1743
1
FLTG01008985.1
0
3688
1
FLTG01013360.1
0
3187
1
FLTG01015373.1
0
1259
1
FLTG01018488.1
0
3218
1

***A. elipticum* Class I RNAs (n=17)**

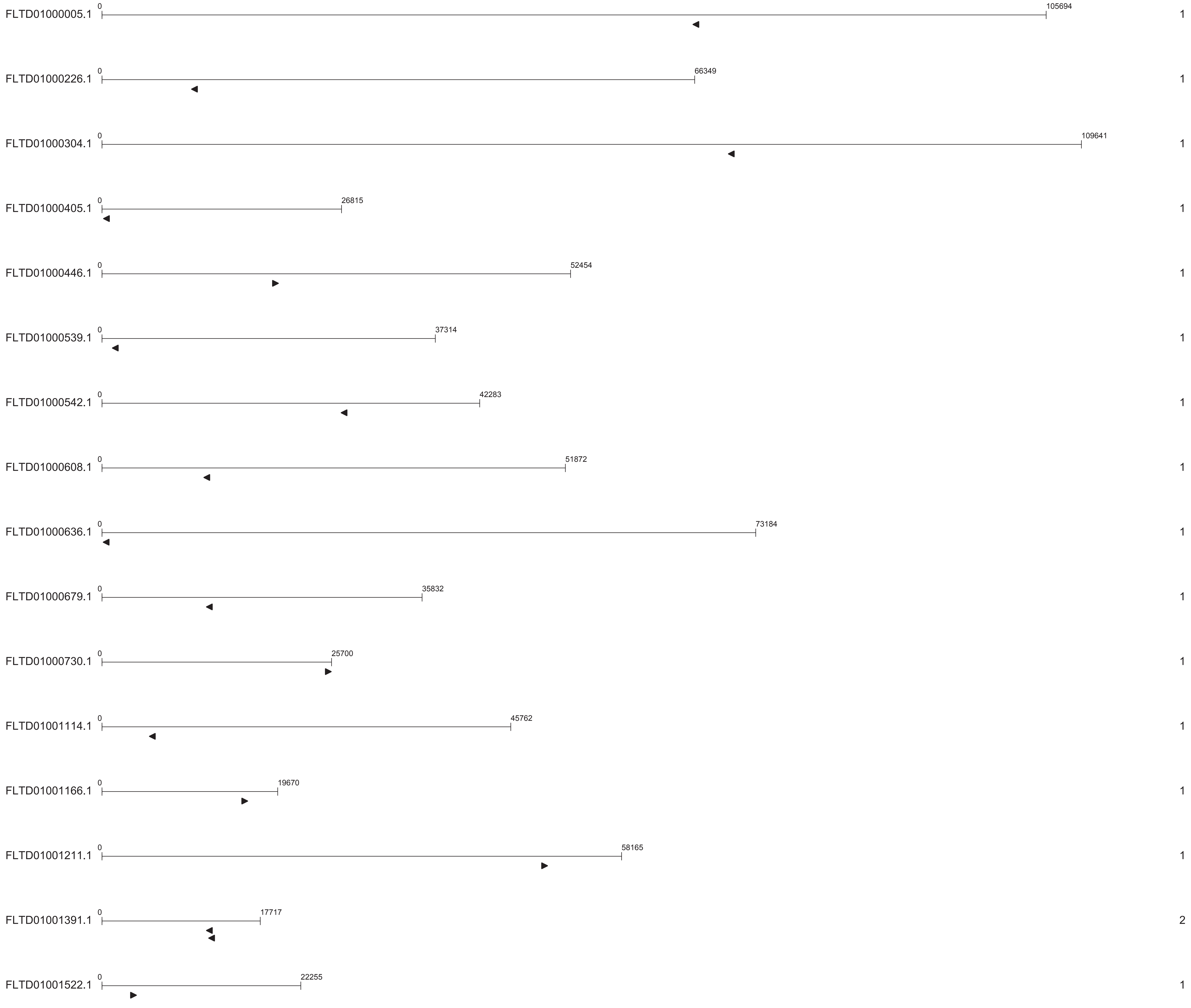

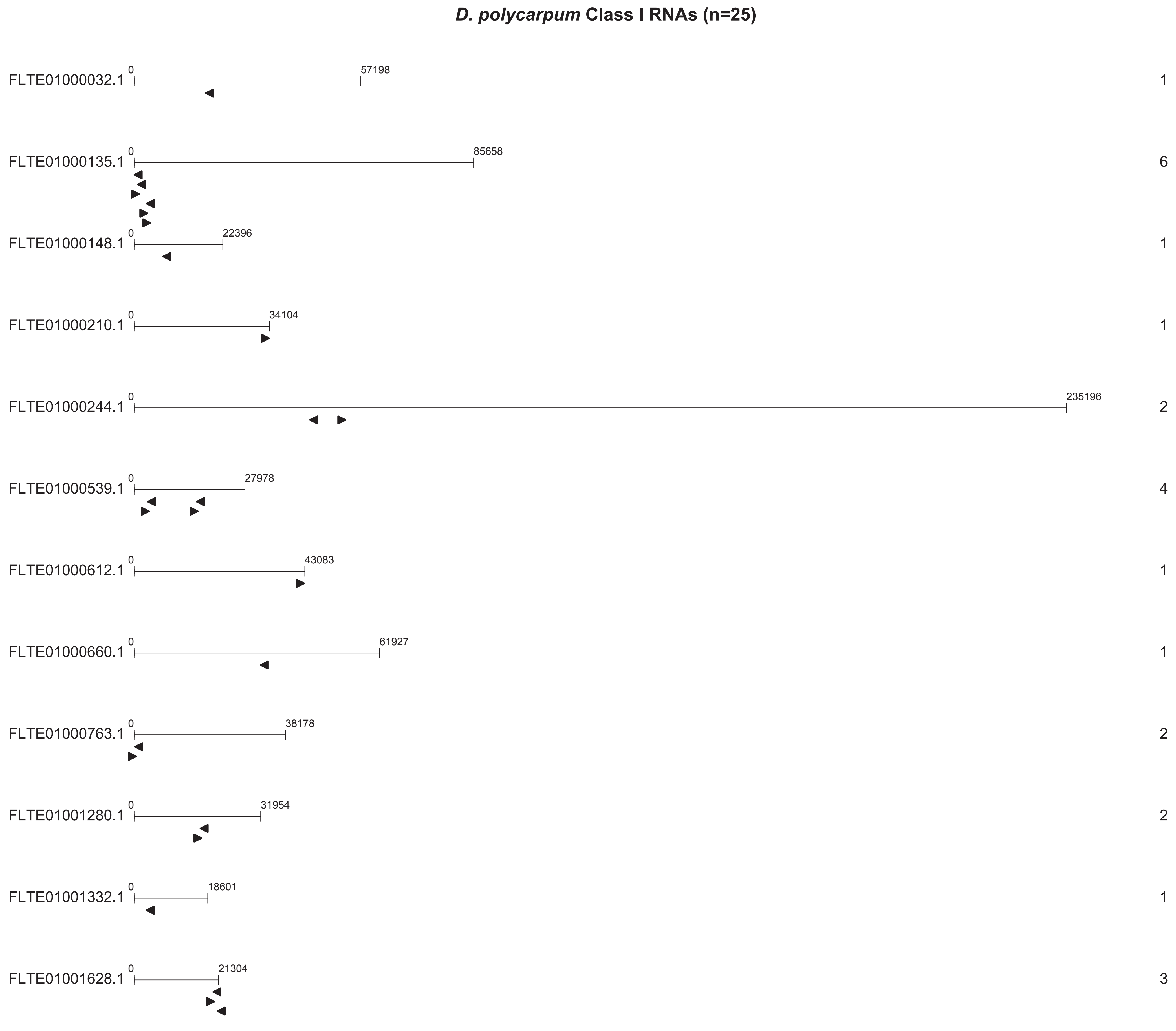

D. polycarpum Class I RNAs (n=25)
FLTE01000032.1
0
57198
1
FLTE01000135.1
0
85658
6
FLTE01000148.1
0
22396
1
FLTE01000210.1
0
34104
1
FLTE01000244.1
0
235196
2
FLTE01000539.1
0
27978
4
FLTE01000612.1
0
43083
1
FLTE01000660.1
0
61927
1
FLTE01000763.1
0
38178
2
FLTE01001280.1
0
31954
2
FLTE01001332.1
0
18601
1
FLTE01001628.1
0
21304
3

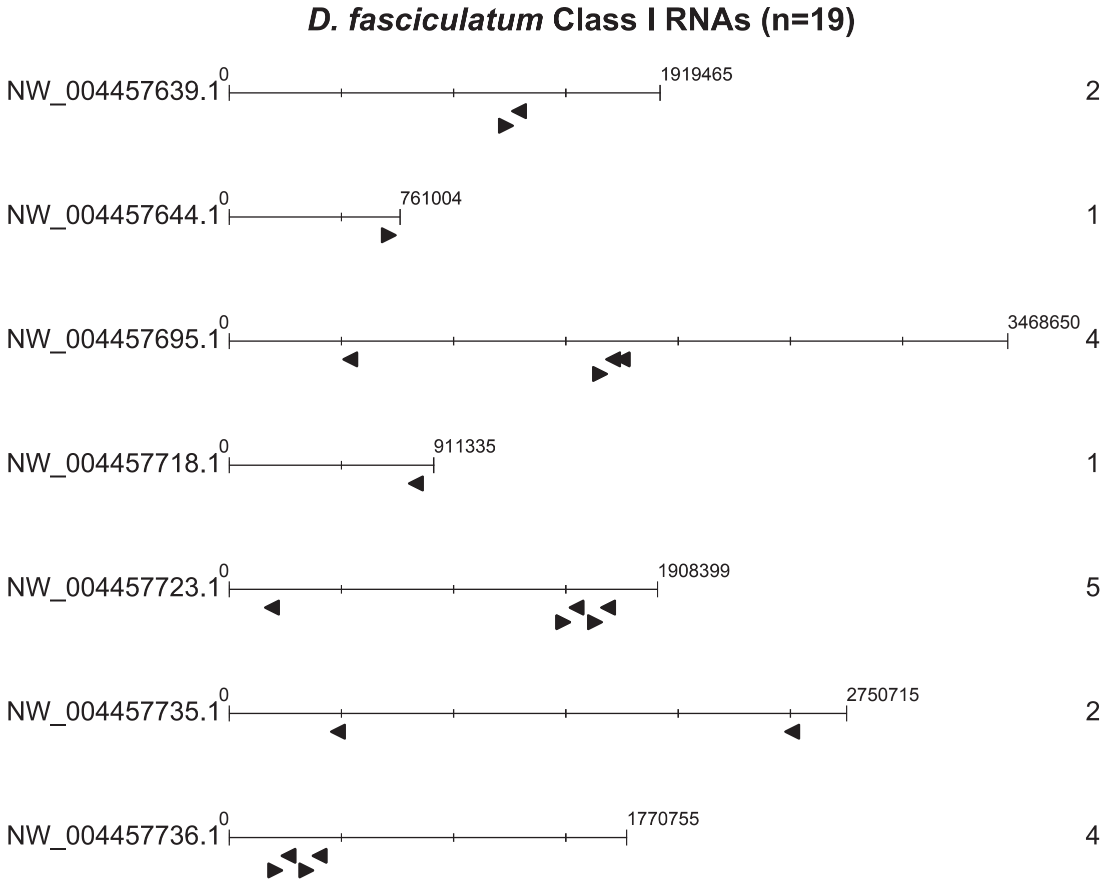
D. fasciculatum Class I RNAs (n=19)
NW_004457639.1
0
1919465
2
NW_004457644.1
0
761004
1
NW_004457695.1
0
3468650
4
NW_004457718.1
0
911335
1
NW_004457723.1
0
1908399
5
NW_004457735.1
0
2750715
2
NW_004457736.1
0
1770755
4

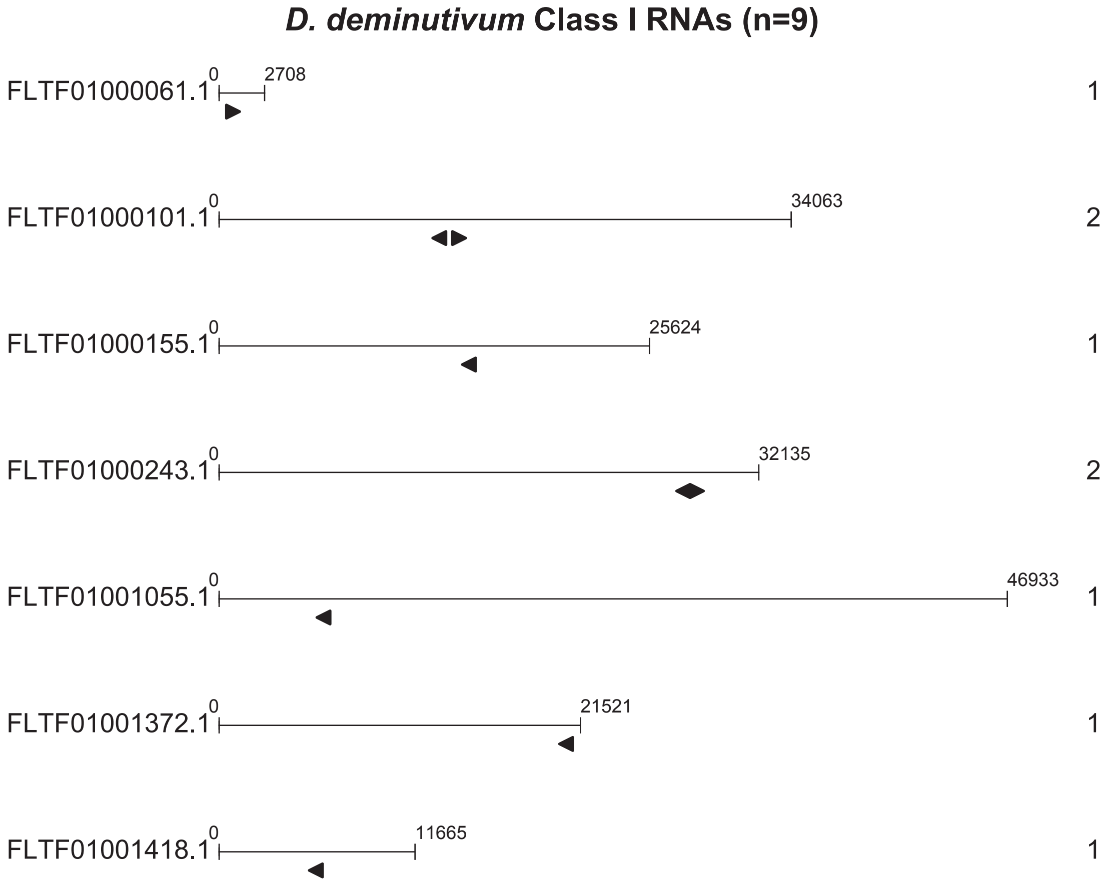
D. deminutivum Class I RNAs (n=9)
FLTF01000061.1
0
2708
1
FLTF01000101.1
0
34063
2
FLTF01000155.1
0
25624
1
FLTF01000243.1
0
32135
2
FLTF01001055.1
0
46933
1
FLTF01001372.1
0
21521
1
FLTF01001418.1
0
11665
1
