# Supplementary material for: Abundantly expressed class of noncoding RNAs conserved through the multicellular evolution of dictyostelid social amoebas
Source: Genome Res. 2021 Mar;31(3):436–47. doi: 10.1101/gr.272856.120 (PMC7919456; doi:10.1101/gr.272856.120)
Supplement: Supplemental Material [file supp_gr.272856.120_Supplemental_Fig_S8.pdf]

Supplemental Figure S8. Shared synteny between *D. discoideum* and *D. firmibasis* Class I RNA loci.

Representation of *D. firmibasis* (dfi) Class I RNA loci +/- 10 kb that share synteny with *D. discoideum* (ddi) Class I RNAs supported by at least two orthologous genes (connected with dashed lines). Chromosome/contig names are given to the left of the genes. Genes situated on the forward and the reverse strand are indicated above and below the chromosome/contig, respectively. Vertical grey lines are given at every 1000 nt.

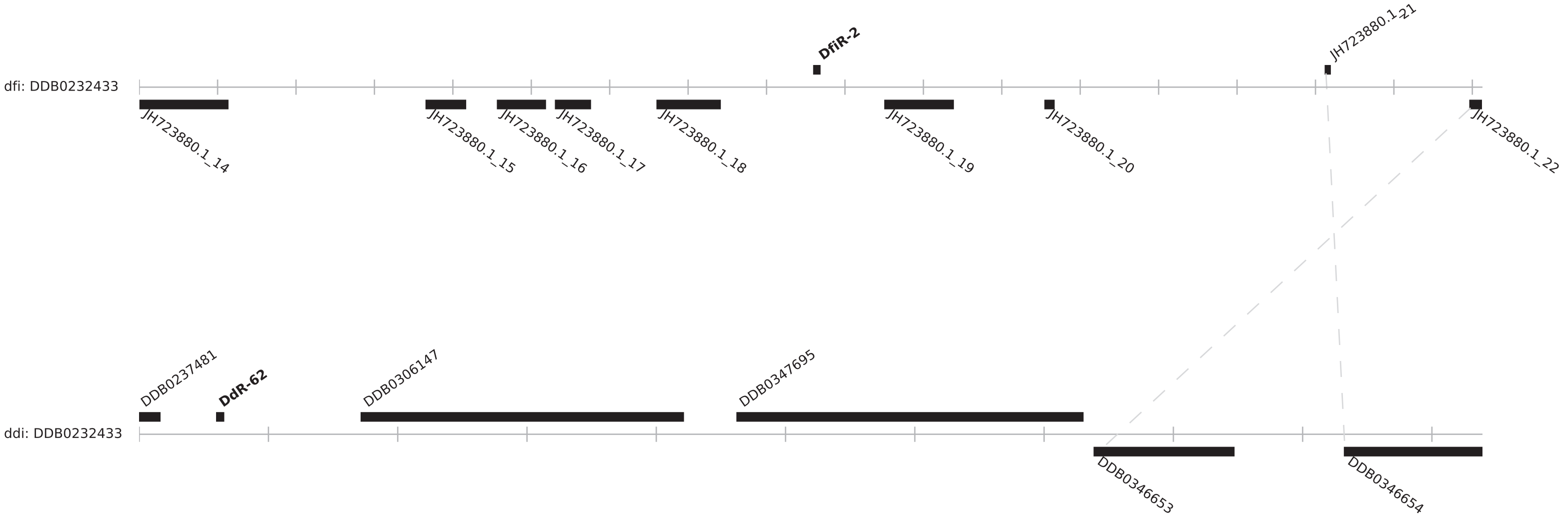

dfi: DDB0232433
DfiR-2
JH723880.1_21
JH723880.1_14
JH723880.1_15
JH723880.1_16
JH723880.1_17
JH723880.1_18
JH723880.1_19
JH723880.1_20
JH723880.1_22
ddi: DDB0232433
DDB0237481
DdR-62
DDB0306147
DDB0347695
DDB0346653
DDB0346654

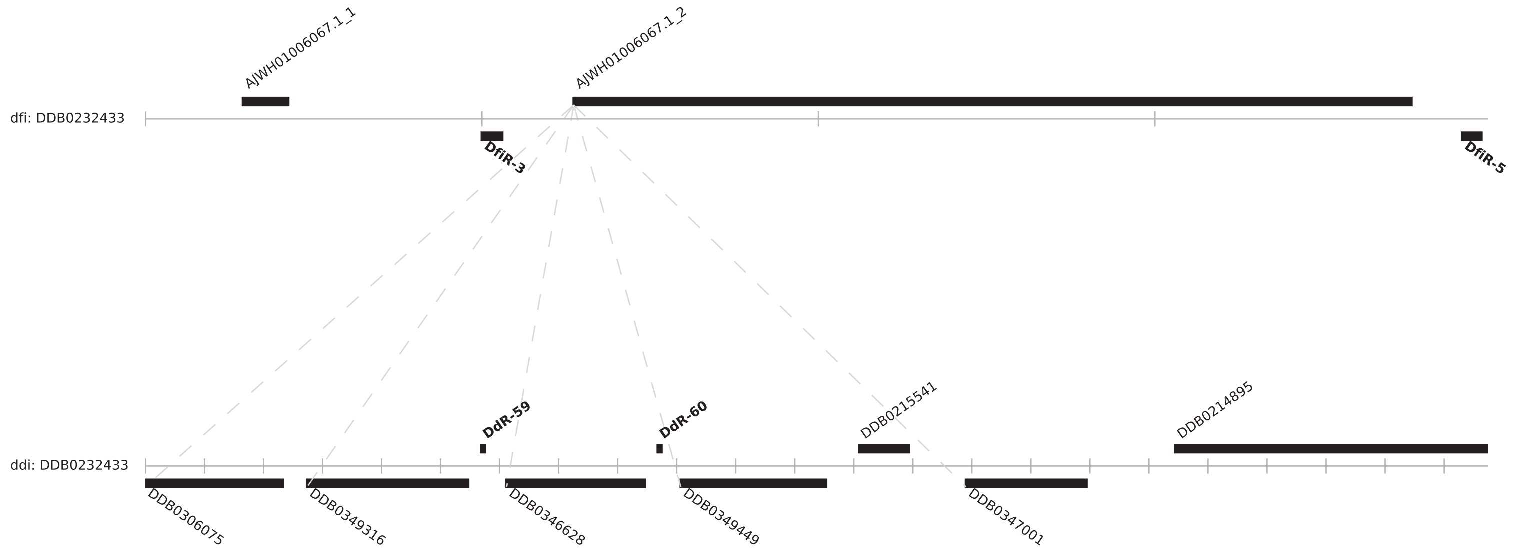

AJWH01006067.1_1
AJWH01006067.1_2
dfi: DDB0232433
DfiR-3
DfiR-5
DdR-59
DdR-60
DDB0215541
DDB0214895
ddi: DDB0232433
DDB0306075
DDB0349316
DDB0346628
DDB0349449
DDB0347001

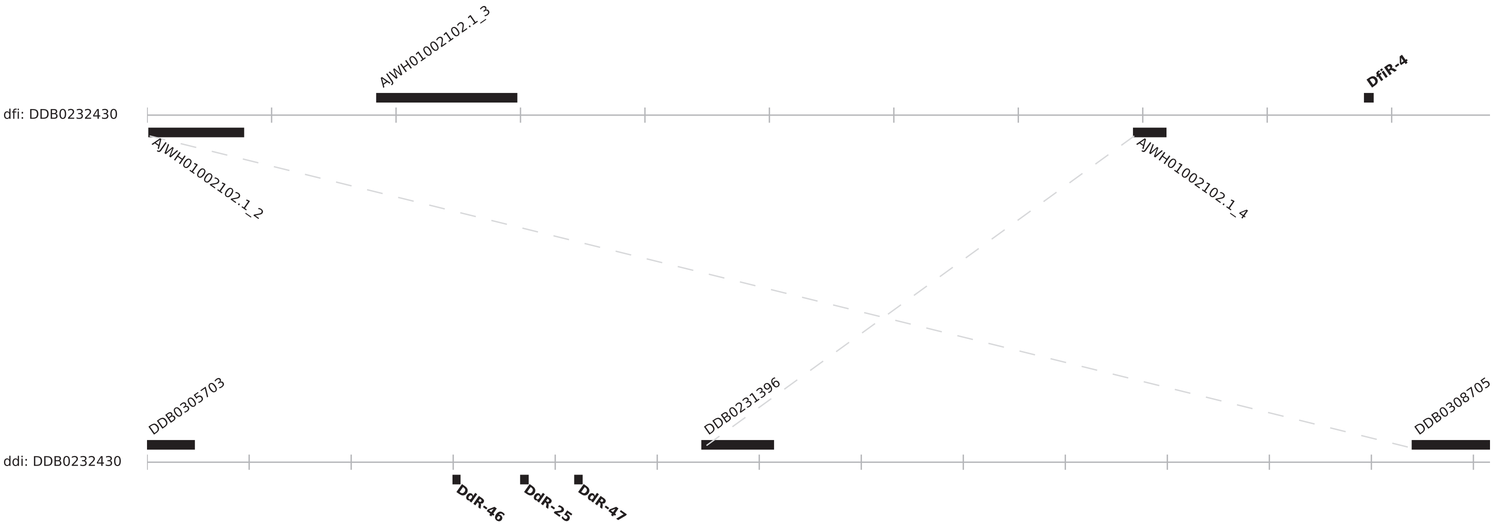

AJWH01002102.1_3
DfiR-4
dfi: DDB0232430
AJWH01002102.1_2
AJWH01002102.1_4
DDB0305703
DDB0231396
DDB0308705
ddi: DDB0232430
DdR-46
DdR-25
DdR-47

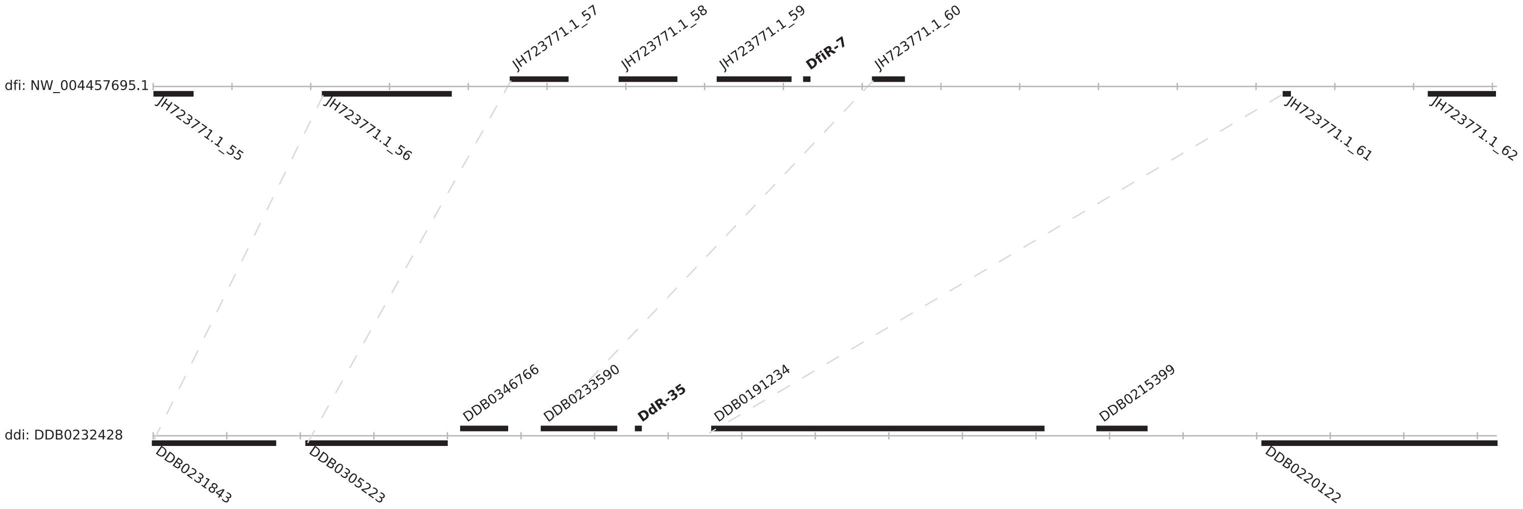

dfi: NW_004457695.1
JH723771.1_57
JH723771.1_58
JH723771.1_59
DfiR-7
JH723771.1_60
JH723771.1_55
JH723771.1_56
JH723771.1_61
JH723771.1_62
ddi: DDB0232428
DDB0346766
DDB0233590
DdR-35
DDB0191234
DDB0215399
DDB0231843
DDB0305223
DDB0220122

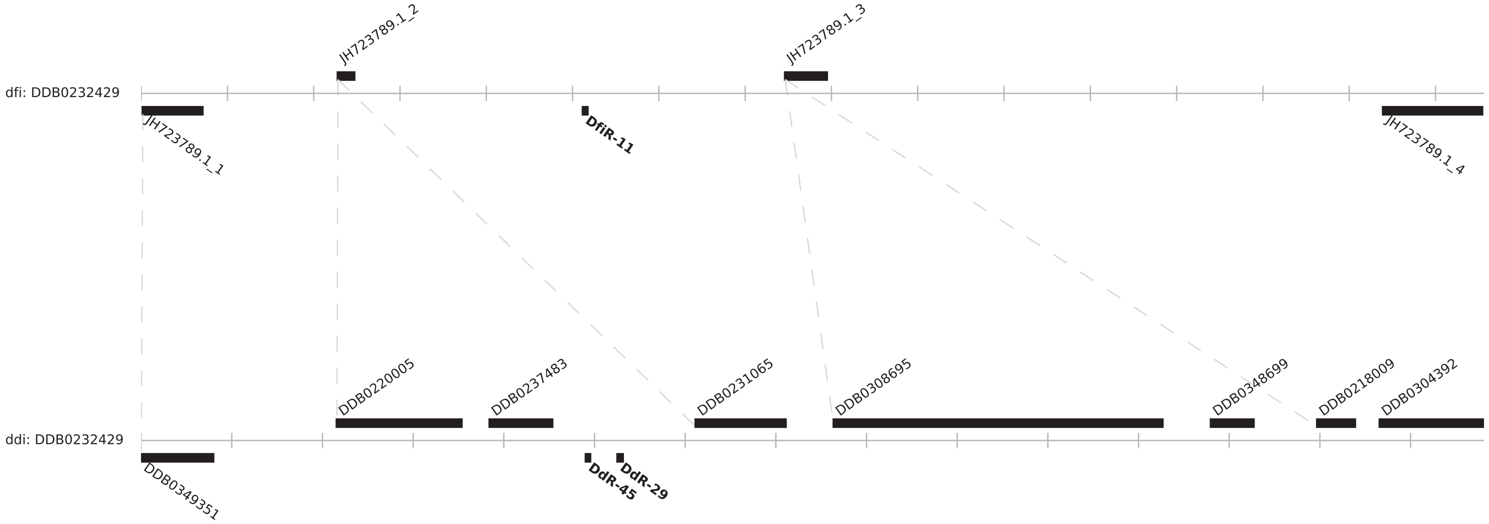

JH723789.1_2
JH723789.1_3
dfi: DDB0232429
JH723789.1_1
DfiR-11
JH723789.1_4
DDB0220005
DDB0237483
DDB0231065
DDB0308695
DDB0348699
DDB0218009
DDB0304392
ddi: DDB0232429
DDB0349351
DdR-45
DdR-29
